# Activation of perfluoro(methyl vinyl ether) at Rh(i) complexes: metal-centered *versus* phosphine-mediated decarbonylation†

**DOI:** 10.1039/d5sc02056e

**Published:** 2025-05-12

**Authors:** Soodeh Mollasalehi, Mike Ahrens, Thomas Braun

**Affiliations:** a Department of Chemistry, Humboldt-Universität zu Berlin Brook-Taylor Str. 2 12489 Berlin Germany thomas.braun@cms.hu-berlin.de

## Abstract

This study investigates the reactivity of perfluoro(methyl vinyl ether) [PMVE, CF_2_=CF(OCF_3_)] towards rhodium(i) phosphine complexes. The reaction pathways are characterized by C–O and C–F bond cleavage reactions and decarbonylation steps. On using the complex [Rh(H)(PEt_3_)_3_] (1), unprecedented reactivity pathways were observed that are distinct from those found for previously studied fluoroolefins. Reactivity of an excess PMVE at Rh is initiated by coordination to the Rh center in 1, followed by its insertion into the Rh–H bond and a β-OCF_3_ elimination. This process ultimately results in OCF_3_ ligand transformation to give *trans*-[Rh(F)(CO)(PEt_3_)_3_] (4) and Et_3_PF_2_. Reactions of stoichiometric amounts of PMVE with [Rh(H)(PEt_3_)_3_] (1) or an excess amount of it with [Rh(F)(PEt_3_)_3_] (6) led to olefin complex formation to yield *trans*-[Rh(F)(η^2^-CF_2_CFH)(PEt_3_)_2_] (7) and *trans*-[Rh(F)(CF(OCF_3_)CF_2_)(PEt_3_)_2_] (8), respectively. In contrast, a remarkable insertion into the Rh–F bond at [Rh(F)(CO)(PEt_3_)_2_] (4) was observed leading to the formation of *trans*-[Rh(CO)(CF(OCF_3_)CF_3_)(PEt_3_)_2_] (5). Decarbonylation of PMVE proceeds not only at Rh, but also *via* a metal-free, phosphine-mediated process. The latter is characterized by oxidative addition of PMVE at PEt_3_ to form the fluorophosphoranes *E*/*Z*-(F_3_CO)CF=CF(PFEt_3_), which subsequently convert into Et_3_PF_2_, CO and presumably tetrafluoroethene.

## Abstract 2

Perfluoro(methyl vinyl ether) serves as CO source and fluorinating agent in the interaction with Rh and phosphine, involving C–O and C–F bond cleavage reactions and decarbonylation steps.

## Graphical abstract

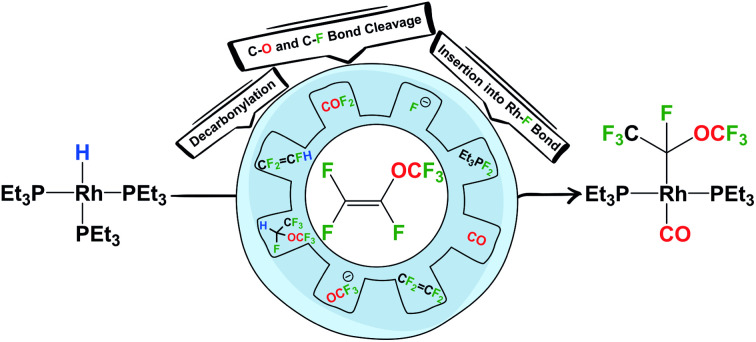

## Introduction

The increasing interest in hydrofluoroolefins (HFOs) stems from their significantly lower potential for ozone depletion and global warming compared to chlorofluorocarbons and hydrofluorocarbons.^1–3^ This has led to their adoption as refrigerants in automotive air conditioning systems.^4–6^ Generally, fluorinated olefins show promise as precursors for synthesizing new fluorinated building blocks.^7–10^ An interesting strategy involves the development of methods for functionalization that are mediated by main group elements or transition metals, including C–F and C–H bond activation reactions.^11–14^

The fluoroolefin perfluoro (methyl vinyl ether) [PMVE, CF_2_=CF(OCF_3_)] can be classified as part of the PFAS family (per- and polyfluoroalkyl substances) and, therefore, understanding its reactivity will provide insights concerning its depletion.^15–17^ It is, however, valuable as a monomer for producing high-value fluorinated polymers.^18–22^ It has also been suggested as an alternative replacement for SF_6_.^23,24^ PMVE is formally an analogue of hexafluoropropene with a formal substitution of the CF_3_ group in hexafluoropropene with the OCF_3_ group, which leads to a different reactivity. Note that the trifluoromethoxy group OCF_3_ imparts significant biological properties to molecules, making OCF_3_ compounds highly valuable targets in the pharmaceutical and agrochemical industries.^25,26^ This is primarily due to the strong electron-withdrawing effect and high lipophilicity caused by this group.^27–32^ To ensure these compounds are used safely in industrial applications, thorough research into their properties and reactivity is essential.^17,33^

Investigations into the reactivity of PMVE towards transition metal complexes are scarce, despite extensive research on fluoroolefins bearing a CF_3_ group. Noticeably, Baker *et al.* previously reported a copper catalysed hydrodefluorination of PMVE leading to the formation of difluoroethylene isomers *via* β-OCF_3_ elimination.^34^ In another recent work, they described a copper-mediated fluoroalkyl –CF(OCF_3_)(CF_2_H) transfer to organic electrophiles *via* insertion of the PMVE into Cu–H bonds of Stryker's reagent.^35^ Note that the hydroamination of PMVE with secondary amines, as well as the addition reaction of azoles to PMVE have been reported.^36,37^

The notable reactivity of rhodium(i) phosphine complexes [Rh(E)(PEt_3_)_3_] (E = H, F, boryl, germyl, silyl) towards fluorinated olefins, such as hexafluoropropene, has been well-documented.^8,38,39^ The identity of the anionic ligand E holds a significant importance in the activation process for several reasons, such as the formation of strong element-fluorine bonds like H–F, B–F, Ge–F, or Si–F bonds, as well as a kinetic control of regio- and chemoselectivities.^8,38–43^

On the other hand, examples of C(sp^2^)–F bond functionalization reactions by simple tertiary phosphines, without the need for activation by metals, metalloids, or a Lewis acid are still rare. Burton and colleagues demonstrated that tertiary phosphines such as PPh_3_ and P^*n*^Bu_3_ could attack perfluorinated cyclic alkenes and perfluorinated linear terminal alkenes leading to the production of stable phosphonium ylides and the generation of a fluorophosphorane ^*n*^Bu_3_P(F)CF=CF(CF_3_), respectively.^44,45^ Additionally, García and colleagues reported the formation of the difluorophosphorane Et_3_PF_2_ in the hydrodefluorination of a range of polyfluoro(hetero)aromatics, using PEt_3_ as the sole defluorinating agent.^46^ Furthermore, the generation of fluorophosphoranes Et_3_P(F)CF=C(X)CF_3_ (X = H, F) or Et_3_PF_2_ through the reaction of various fluorinated olefins with the [Rh(E)(PEt_3_)_3_] has been reported.^38,41,47^ Dissociation of phosphine during the C–F bond activation step at the Rh complex might be essential for these transformations.

Herein we present studies on the reactivity of PMVE towards the rhodium(i) phosphine complexes [Rh(H)(PEt_3_)_3_] (1) and [Rh(F)(PEt_3_)_3_] (6). PMVE serves as CO source and fluorinating agent. The observed reactivity patterns are very distinctive and involve coordination of the olefin, insertion into Rh–H and Rh–F bonds, β-OCF_3_ elimination steps as well as oxidative addition at liberated PEt_3_.

## Results and discussion

**Scheme 1:**
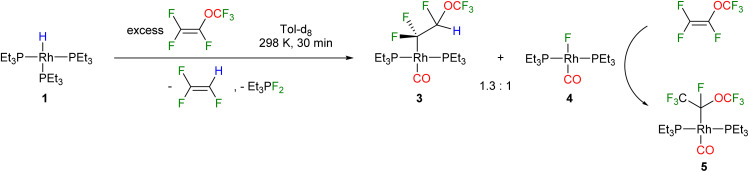
Reactivity of complex 1 towards PMVE.

Treatment of the rhodium(i) hydrido complex [Rh(H)(PEt_3_)_3_] (1) with an excess of perfluoro (methyl vinyl ether) [PMVE, CF_2_=CF(OCF_3_)] yielded after 30 minutes the fluoroalkyl complex *trans*-[Rh(CF_2_CFH(OCF_3_))(CO)(PEt_3_)_2_] (3) and the Vaska type fluorido complex *trans*-[Rh(F)(CO)(PEt_3_)_2_] (4) in a ratio of 1.3 : 1, as well as 1,1,2-trifluoroethylene (Scheme 1). The source of the CO ligand in 3 and 4 is the OCF_3_ moiety of PMVE, and its rhodium as well as phosphine mediated-transformation is described below.

The compounds 3 and 4 were not stable at room temperature and a slow conversion into the rhodium(l) perfluoroalkyl complex *trans*-[Rh(CO)(CF(OCF_3_)CF_3_)(PEt_3_)_2_] (5) was found (Scheme 1). The formation of complex 5 suggests an insertion of PMVE, which is present in an excess, into the rhodium–fluorine bond in complex 4. An independent reaction of 4 with PMVE confirmed the formation of 5*via* the insertion of the olefin into the Rh–F bond. Note that examples for an insertion of fluorinated olefins into metal–fluorine bonds are rare, and have been reported previously at group 10 and 11 transition metal-complexes, but not at rhodium.^35,48,49^

Compound 4 has been previously synthesised and fully characterised.^50^ Complexes 3 and 5 were characterised by ^1^H, ^31^P{^1^H} and ^19^F NMR spectroscopy. For complex 3, in the ^31^P{^1^H} spectrum a doublet of triplets is observable due to the coupling to the rhodium centre (138.3 Hz) and the CF_2_ moiety (24.4 Hz). In the ^19^F NMR spectrum, the resonance of the OCF_3_ moiety appears at −58.4 as a broad signal, while for the CF_2_ group two resonances were found with a geminal F,F coupling constant of 295 Hz, which is in accordance with the C(sp^3^) character of the CF_2_ group.^34,51^ For the CFH group a doublet with ^2^*J*_F,H_ of 57 Hz at −134.9 ppm in ^19^F NMR spectrum and at 5.8 ppm in ^1^H NMR spectrum are visible. The IR spectrum of 3 reveals an intense absorption band at 1945 cm^−1^, which can be assigned to the CO stretching vibration and the data are comparable with those of known rhodium carbonyl complexes.^50^ For complex 5 on the other hand, a doublet appears in ^31^P{^1^H} NMR spectrum at 24.3 ppm with a P,Rh coupling constant of 120.9 Hz. In the ^19^F NMR spectrum, a doublet of quartets at −53.7 ppm corresponds to the CF_3_ moiety, which couples to the CF group with 10 Hz and to the OCF_3_ group with 1 Hz, while another doublet of quartets at −81.5 ppm with a coupling constant of 3 Hz to the CF group can be assigned to the OCF_3_ moiety. Both signals correlate with the quartet of quartets at −129.5 ppm for the CF moiety. Furthermore, a CO stretching vibration band at 1938 cm^−1^ in the IR spectrum is observable for compound 5.

**Scheme 2:**
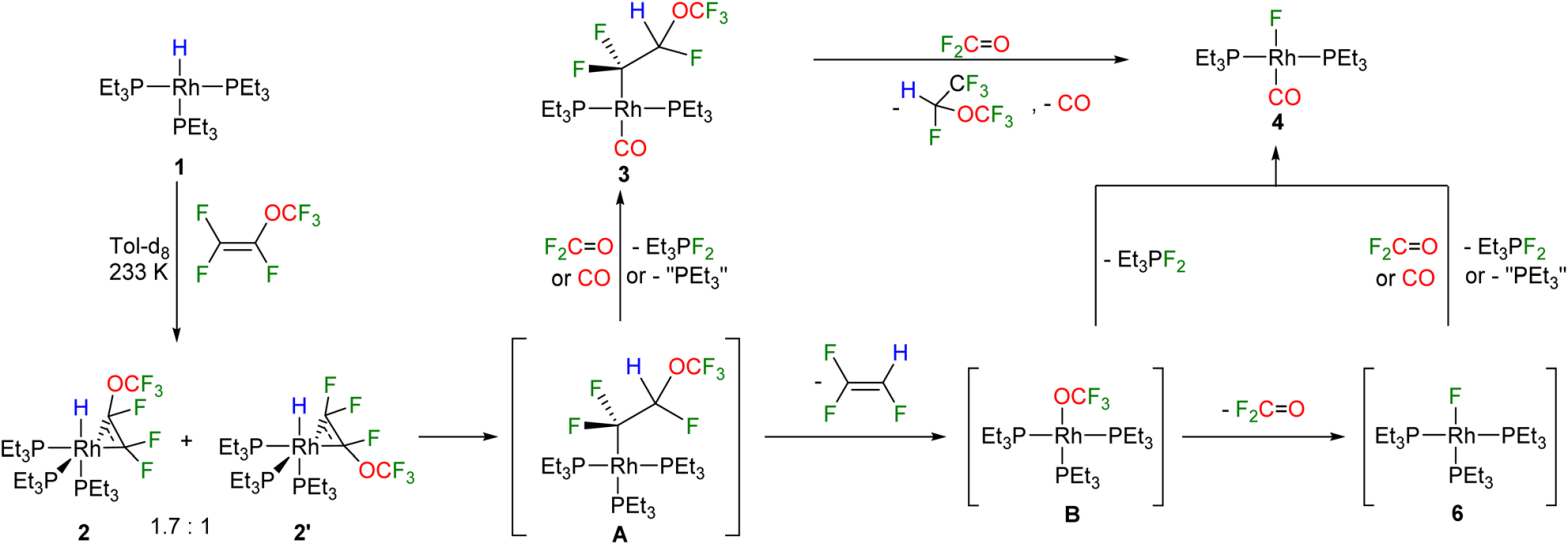
Mechanistic proposal for the reactivity of complex 1 towards PMVE.

Moreover, the formation of the fluorophosphorane Et_3_PF_2_ and 1,2,2,2-tetrafluoroethyl trifluoromethyl ether, CHF(OCF_3_)(CF_3_) was observed (see also below, Scheme 2).^47^ The presence of CHF(OCF_3_)(CF_3_) was revealed by the ^19^F and ^1^H NMR spectra data, which were in accordance with the literature.^52^

**Fig. 1:**
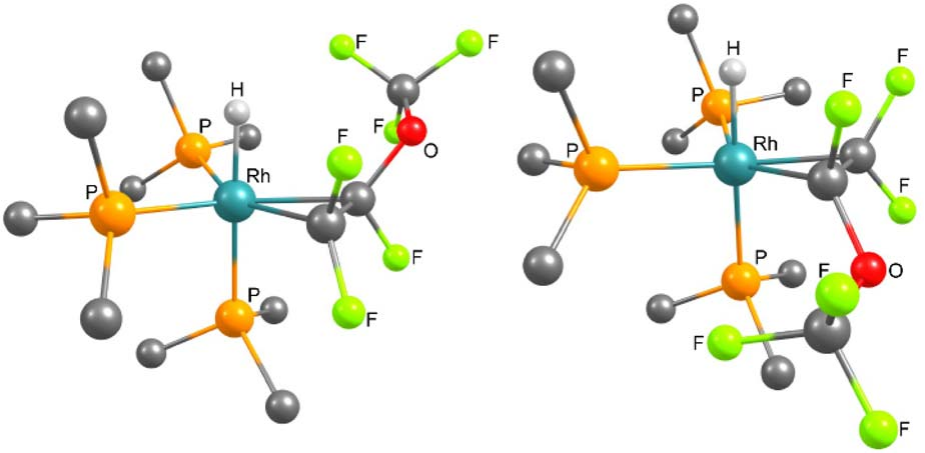
DFT optimised structures ((B3LYP/cc-pvtz and cc-pvdz), see ESI†) of left: *fac*-[Rh(H){η^2^-CF_2_=CF(OCF_3_)}(PMe_3_)_3_] (2*), and right: (2′*). Hydrogen atoms of the phosphine ligands are omitted for clarity.

The reaction of 1 with PMVE was then monitored by NMR spectroscopy at 233 K and the isomeric rotational intermediates *fac*-[Rh(H)(η^2^-CF_2_=C(OCF_3_)F)(PEt_3_)_3_] (2/2′) in a ratio of 1.7 : 1 were identified (Scheme 2). These olefin complexes were only stable below 273 K, therefore, identification was possible by ^1^H, ^31^P{^1^H} and ^19^F NMR spectroscopy. Another identified intermediate at this temperature was a fluorophosphorane, *Z*-(F_3_CO)CF=CF(PFEt_3_), which indicates the reaction of PMVE with PEt_3_ (for further explanation see below). Above 273 K the transformation of this compound together with 2 and 2′ into 3, 4, trifluoroethylene, CHF(OCF_3_)(CF_3_), and Et_3_PF_2_ was observed. Previously, coordination of fluorinated olefins to [Rh(H)(PEt_3_)_3_] (1) at low temperatures has been described.^38,43,53^ Characteristic features in the ^1^H NMR spectrum are the signals for the hydrido ligands at −12.1 ppm with a large H,P_*trans*_ coupling constant of 152.3 Hz for 2 and at −11.9 ppm for 2′ with 152.8 Hz. Additionally, in the ^1^H{^31^P} NMR spectrum, a doublet of doublets appears for 2 due to the coupling to Rh and the one fluorine atom in *syn* position, while for 2′ a doublet of triplets due to the coupling to Rh and two fluorine atoms in *syn* position is visible. Due to the high structural similarities of the two isomers, the three sets of signals for the inequivalent phosphine ligands at 14.0–12.8 ppm, 11.2–9.4 ppm and 4.8–2.6 ppm, which integrate in a 1 : 1 : 1 ratio, overlap in the ^31^P{^1^H} NMR spectrum (242 MHz) at 273 K. However, the Rh,P coupling constants are in accordance with the oxidation state +III at rhodium and a metallacyclopropane structure.^38,43^ In order to further support the metallacyclopropyl configuration of the complexes 2 and 2′, the structures of the corresponding PMe_3_ derivatives 2* and 2′* were optimized by DFT calculations and a C–C bond distance of 1.472 Å for 2* and 2′* was obtained (Fig. 1).^54–56^ Calculations on the PEt_3_ complexes 2 and 2′ were not conclusive as the energy differences of the converged structures were highly dependent on slight structural differences in the PEt_3_ ligand sphere, thus making it hard to identify the global minima. However, based on the DFT calculations on the simplified PMe_3_ derivatives, complex 2* is more stable than complex 2′* by 10.7 kJ mol^−1^.^54–56^

Mechanistically, the transformation of intermediates 2 and 2′ into complexes 3 and 4 requires an insertion of the olefin into the Rh–H bond to yield the intermediate A (Scheme 2). A then undergoes a β-OCF_3_ elimination accompanied by the release of the trifluoroethylene. It is likely that the complex [Rh(OCF_3_)(PEt_3_)_3_] (B) is then formed as an unstable intermediate, which converts into 4 and F_2_PEt_3_. Alternatively, the fluorido complex [Rh(F)(PEt_3_)_3_] (6) and fluorophosgene O=CF_2_ are generated. The rapid decomposition of a trifluoromethoxide anion OCF_3_^−^ into fluorophosgene O=CF_2_ and fluoride has been reported in literature.^31^ Fluorophosgene can react with A or 6 to yield Et_3_PF_2_ as well as the carbonyl complexes 3 and 4, respectively. In addition, it is also feasible that complex 3 reacts with fluorophosgene to convert into 4 upon the release of the observed CHF(OCF_3_)(CF_3_) and CO (Scheme 2). Note that olefin insertion into the metal–hydrogen bond and β-OCF_3_ elimination steps have been proposed for a copper complex that mediates the catalytic hydrodefluorination of PMVE to yield a mixture of *cis*- and *trans*-1,2-difluoroethylene in a 4 : 1 ratio.^34^

**Scheme 3:**
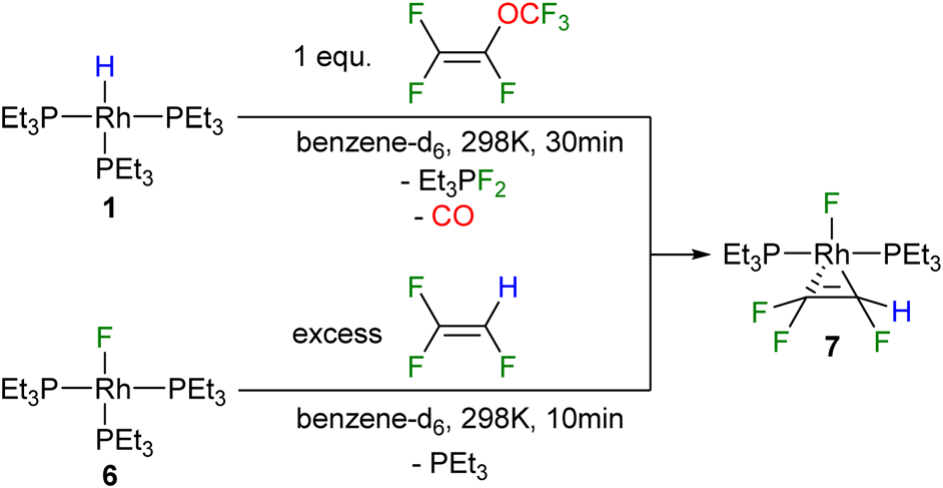
Reactivity of complex 1 towards one equivalent of PMVE (top), and reactivity of complex 6 towards trifluoroethylene (bottom).

Furthermore, it is important to consider that for the conversion of A into 3 and for the formation of the fluorido complex 4 from [Rh(F)(PEt_3_)_3_] (6) at least two equivalents of PMVE as an excess source of CO are required. Hence, initially the role of olefin was investigated further by the reaction of [Rh(H)(PEt_3_)_3_] (1) with stoichiometric amounts of PMVE at room temperature, which led to the formation of *trans*-[Rh(F)(η^2^-CF_2_CFH)(PEt_3_)_2_] (7) as well as the fluorophosphorane Et_3_PF_2_ (Scheme 3). Formation of 7 implies the generation of the reaction products as described above, whereas subsequently a phosphine ligand is substituted by trifluoroethylene at [Rh(F)(PEt_3_)_3_] (6) (Scheme 2). The latter step was confirmed independently by treatment of the rhodium fluorido complex [Rh(F)(PEt_3_)_3_] (6) with trifluoroethylene to yield complex 7 (Scheme 3). Thus, the result demonstrates that a replacement of the phosphine ligand in [Rh(F)(PEt_3_)_3_] (6) by an olefin can compete with the substitution of the phosphine by CO, when less amounts of PMVE as a CO source are provided. Note that the dissociation of a phosphine ligand upon the coordination of a fluoroolefin to the fluorido complex 6 has been previously reported for 1,1,3,3,3-pentafluoropropene in a similar manner.^38^

The resonances of the two inequivalent phosphine ligands of complex 7 appear at 32.3 and 25.9 ppm in the ^31^P{^1^H} NMR spectrum, and are coupled to each other by *trans*^2^*J*_p,p_ = 368.2 Hz. The phosphorus–rhodium coupling constants of 138.9 Hz and 133.3 Hz, respectively, demonstrate the presence of a Rh(i) complex. The signal at 25.9 ppm represents a doublet of doublet of doublet of doublets due to its coupling to phosphorus, rhodium, one fluorine atom of the CF_2_ moiety and the fluorido ligand, while the second signal displays a doublet of doublet of triplets due to couplings to phosphorus and rhodium, as well as coupling to one fluorine atom of the CF_2_ moiety and the fluorine atom of the CFH group. In the ^19^F NMR spectrum, a geminally coupled pair is observed at −89.4 and −90.8 ppm (^2^*J*_F,F_ = 109 Hz) associated with the CF_2_ fluorine resonances along with an upfield CFH peak at −194.9 ppm and the signal of the fluorido ligand at −218.3 ppm. A distinctive doublet of doublet of doublets appears at 5.5 ppm in the ^1^H NMR spectrum, corresponding to the CFH hydrogen atom (^2^*J*_FH_ = 73.3 Hz).

**Scheme 4:**
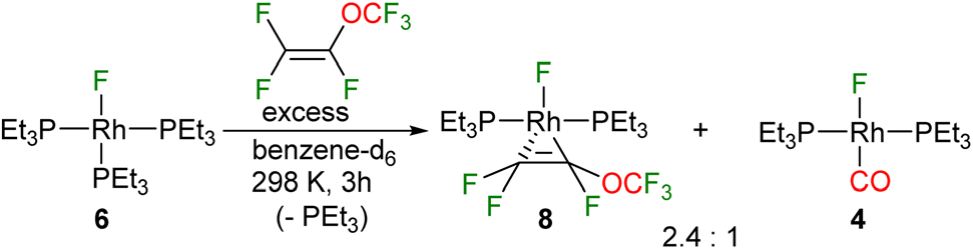
Reactivity of complex 6 towards PMVE.

The reactivity described above of the fluorido complex [Rh(F)(CO)(PEt_3_)_2_] (4) towards PMVE (Scheme 1) prompted us to also study the behaviour of the fluorido compound [Rh(F)(PEt_3_)_3_] (6) towards PMVE, in order to assess a possible coordination *versus* insertion in a Rh–F bond, as the latter step was found for [Rh(F)(CO)(PEt_3_)_2_] (4). However, treatment of 6 with PMVE gave after 3 hours a mixture of complexes [Rh(F)(η^2^-CF(OCF_3_)CF_2_)(PEt_3_)_2_] (8) and 4 in a 2.4 : 1 ratio, and the Et_3_PF_2_ (Scheme 4). At the beginning of this reaction the phosphorane *Z*-(F_3_CO)CF=CF(PFEt_3_) was present, but there was no indication for the formation of complex 4. The latter was formed upon depletion of the phosphonium salt indicating a crucial role of *Z*-(F_3_CO)CF=CF(PFEt_3_) as an alternative CO source (see below). It can be presumed that initially *Z*-(F_3_CO)CF=CF(PFEt_3_) was generated by reaction of liberated PEt_3_ with PMVE, which indicates that a dissociation of phosphine takes place and that any rebinding process is slower than the reactivity of the free phosphine towards the excess of olefin. Note that when 6 was treated with PMVE in the presence of oxygen only *trans*-[Rh(F)(η^2^-CF(OCF_3_)CF_2_)(PEt_3_)_2_] (8) was generated as the liberated phosphine can be trapped as Et_3_P=O.

**Fig. 2:**
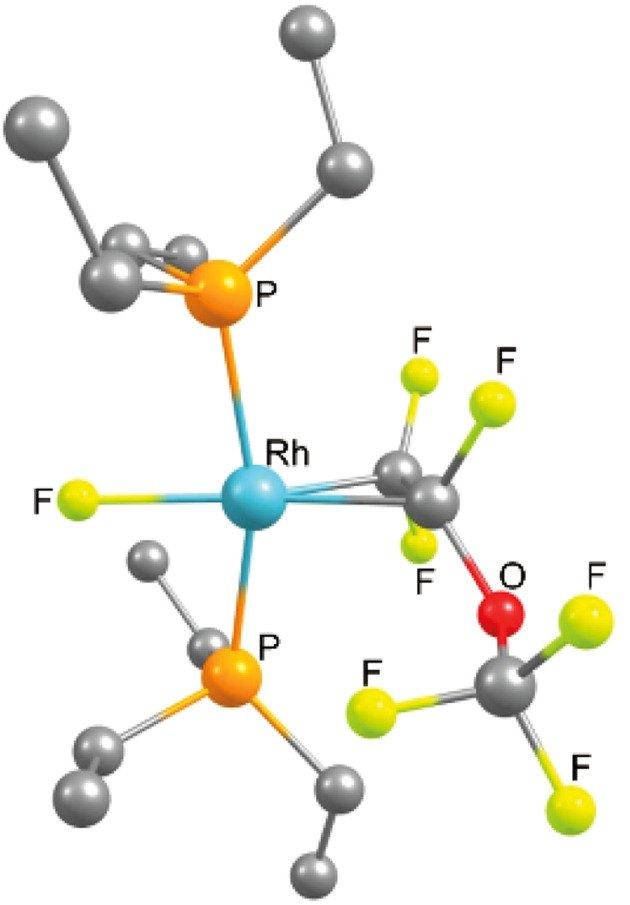
DFT optimised structure (B3LYP/cc-pvdz) of complex 8. Hydrogen atoms of the phosphine ligands are omitted for clarity.

The ^31^P{^1^H} NMR spectrum (202 MHz) of complex 8 displayed two resonances for inequivalent phosphorus atoms at 34.1 and 30.5 ppm. Both signals show a *trans* P–P coupling of 366.7 Hz and P–Rh coupling constants typical of Rh(i) complexes (133.4 and 134.1 Hz). The signal at 30.5 ppm appears as a doublet of doublet of doublet of doublets due to the couplings to phosphorus and rhodium as well as coupling to one of the fluorine atoms at the CF_2_ moiety and the fluorido ligand. In contrast, the second resonance shows a doublet of doublet of doublet of triplet of doublets due to the additional coupling to the CF moiety. In the ^19^F NMR spectrum, five inequivalent resonances for the OCF_3_ moiety at −57.1 ppm, the two fluorine atoms of the CF_2_ group at −96.8 and −98.2 ppm, the CF group at −116.1 ppm and the rhodium bound fluorido ligand at −206.3 ppm are displayed. A correlation between the olefinic fluorine signals and fluorido ligand was confirmed by ^19^F,^19^F-COSY NMR spectroscopy. The C–C bond distance of the olefin obtained by DFT calculations (1.44 Å) suggests a metallacyclopropane configuration (Fig. 2).^54–56^ Note that the NMR as well as the DFT calculation results are in agreement with those that have been previously reported for 1,1,3,3,3-pentafluoropropene.^38^

**Scheme 5:**
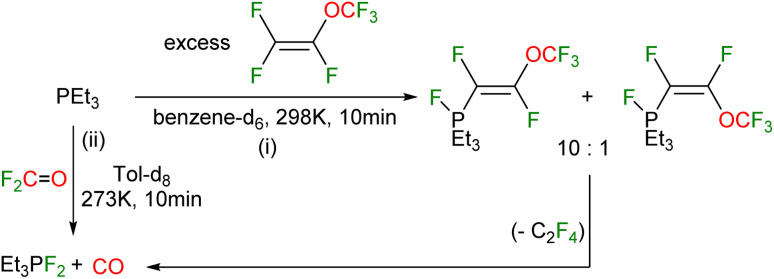
Reactivity of triethylphosphine towards: (i) perfluoro (methyl vinyl ether), and (ii) fluorophosgene.

Independent reactivity studies on the reactivity of PEt_3_ towards PMVE demonstrated that *Z*/*E*-(F_3_CO)CF=CF(PFEt_3_) in a 10 : 1 ratio can be formed by an oxidative addition at the phosphorus center (Scheme 5). A similar reactivity pattern of phosphines with other fluoroolefins, for instance by Burton and Röschenthaler, or fluoroaromatics was previously reported.^38,41,45,47,57,58^ Monitoring the reaction solution after the generation of *Z*/*E*-(F_3_CO)CF=CF(PFEt_3_) at room temperature revealed a conversion into F_2_PEt_3_, CO, and presumably C_2_F_4_ along with other decomposition products. The latter could not be identified, possibly because of oligomerization. The formation of the CO gas was confirmed by ^13^C{^1^H} NMR spectroscopy as well as gas chromatography. In the ^31^P{^1^H} NMR spectrum of *Z*/*E*-(F_3_CO)CF=CF(PFEt_3_) two signals at −63.8 ppm with a ^1^*J*_P,F_ coupling constant of 598.8 Hz and 592.2 Hz are visible. The corresponding signals in the ^19^F NMR appeared at −19.6 and −22.3 ppm for the *Z* and *E* isomers, respectively. Additionally, resonances for the OCF_3_ moiety and the two CF groups with a ^3^*J*_F,F_^*trans*^ of 116 Hz (for the *Z* isomer) and a ^3^*J*_F,F_^*cis*^ of 25 Hz (for the *E* isomer) are displayed.

**Scheme 6:**
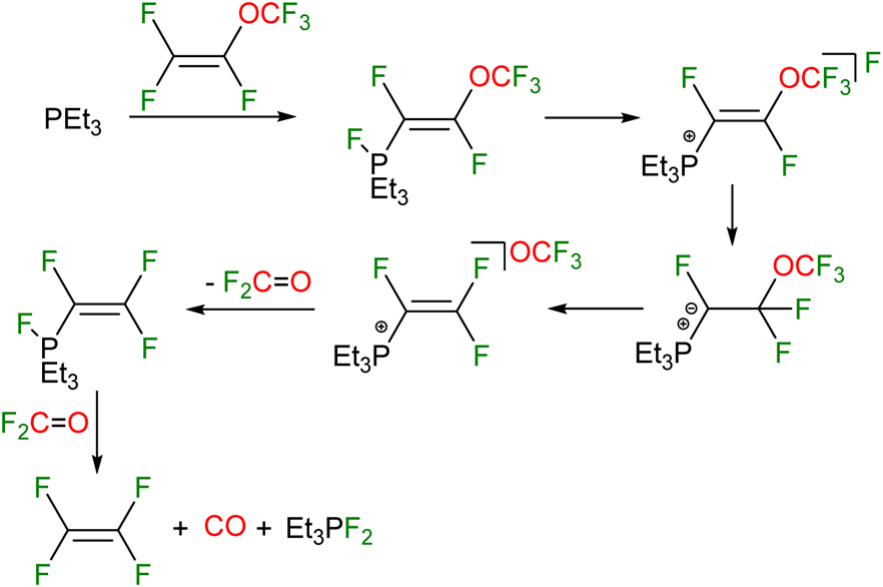
Mechanistic proposal for the decomposition of the fluorophosphorane *Z*-(F_3_CO)CF=CF(PFEt_3_).

Mechanistically it can be assumed that (F_3_CO)CF=CF(PFEt_3_) converts initially into the phosphonium salt (F_3_CO)CF=CF(PEt_3_)^+^F^−^ (Scheme 6). A nucleophilic attack of the fluoride at the vinyl carbon yields the ylide CF_2_(OCF_3_)CF(PEt_3_). Then CF_2_=CF(PFEt_3_) and O=CF_2_ might be generated *via* CF_2_=CF(PEt_3_)^+^OCF_3_^−^. The former CF_2_=CF(PFEt_3_) might convert into F_2_PEt_3_, CO, and C_2_F_4_. Note that an independent reaction shows that F_2_PEt_3_ and CO can also obtained by the reaction of the phosphine with fluorophosgene (Scheme 5).^59^

## Conclusions

The paper demonstrates that the rhodium(i) hydrido complex [Rh(H)(PEt_3_)_3_] (1) exhibits distinct reactivity pathways towards PMVE when compared to the reactivity of other fluorinated olefins. These pathways lead to metal- and phosphine-mediated decarbonylation reactions. Both processes involve the generation of a OCF_3_^−^ moiety that can convert into CO, fluoride and fluorophosgene. While at Rh trifluoroethylene is generated, the phosphine-mediated process presumably leads to the formation of tetrafluoroethene. The studies provide valuable insights into the reactivity of PMVE which is considered a PFAS.^15^ Decarbonylation processes allow for its decomposition and open up new avenues for transforming fluorinated fragments. Remarkably, It is shown that the formation of the rhodium carbonyl fluorido complex 4 allows the insertion of PMVE into the Rh–F bond forming the –CF(OCF_3_)(CF_3_) ligand. Additionally, this paper highlights that phosphines might be suitable tools for a transformation of fluorinated compounds.^14^

## Data availability

Details of the experimental procedures, characterization of the complexes and DFT calculations can be found in the ESI.†

## Author contributions

Conceptualization, S. M. and T. B.; investigation, S. M. and M. A.; writing – original draft preparation, S. M.; writing – review and editing, S. M. and T. B.; funding acquisition, T. B.

## Conflicts of interest

There are no conflicts to declare.

## Supplementary Material

SC-OLF-D5SC02056E-s001
