# Supplementary material for: Activation of perfluoro(methyl vinyl ether) at Rh(i) complexes: metal-centered versus phosphine-mediated decarbonylation
Source: Chem Sci. 2025 May 12;16(24):10928–34. doi: 10.1039/d5sc02056e (PMC12086712; doi:10.1039/d5sc02056e)
Supplement: SC-016-D5SC02056E-s001 [file SC-016-D5SC02056E-s001.pdf]

Supplementary Information (SI) for Chemical Science.
This journal is © The Royal Society of Chemistry 2025

Supporting Information

Activation of Perfluoro(methyl vinyl ether) at Rh(I) Complexes: Metal-Centered versus Phosphine Mediated Decarbonylation

Soodeh Mollasalehi,[a] Mike Ahrens [a] and Thomas Braun [a,*]

[a] *Department of Chemistry, Humboldt-Universität zu Berlin, Brook-Taylor Str. 2, 12489 Berlin (Germany). E-mail: thomas.braun@cms.hu-berlin.de.*

Table of Contents

1.	Synthesis and characterization of all compounds	3
1.1	General Procedures, Methods and Materials	3
1.2	Reaction of PMVE with [Rh(H)(PEt_3)$_3$] (**1**)	3
1.3	Independent reaction of PMVE with [Rh(F)(CO)(PEt_3)$_2$] (**4**)	5
1.4	Reaction of PMVE with triethylphosphine PEt_3	6
1.5	Independent reaction of fluorophosgene with triethylphosphine PEt_3	7
1.6	Reaction of 1 equivalent of the PMVE with [Rh(H)(PEt_3)$_3$] (**1**)	7
1.7	Independent reaction of trifluoroethylene with [Rh(F)(PEt_3)$_3$] (**6**)	7
1.8	Reaction of PMVE with [Rh(F)(PEt_3)$_3$] (**6**)	8
1.9	NMR Spectra	9
2.	DFT Calculations	28
2.1	Computational details for geometry optimization of all the calculated complexes	28
2.2	Structure optimization of complexes **2***, **2’*** and **8**	28
3.	References	35

1. Synthesis and characterization of all compounds

1.1 General Procedures, Methods and Materials

All experiments were carried out under an atmosphere of argon by Schlenk techniques. Solvents were dried by conventional methods[1] and, prior to use, distilled under argon. The rhodium complexes $[Rh(H)(PEt_3)_3]$ (**1**)[2], $[Rh(F)(CO)(PEt_3)_2]$ (**4**)[3] $[Rh(F)(PEt_3)_3]$ (**6**)[4] were prepared as described in the literature. All other reagents were obtained from commercial sources. The mass of gaseous compounds such as perfluoro(methyl vinyl ether), difluorophosgene or trifluoroethylene was determined by condensation into a dried Young NMR tube. The NMR tube was weighed before and after the condensation procedure. The gas was then condensed into a second tube containing the reaction solution by trap-to-trap condensation. In addition, the obtained partial pressure of the gas relates to a certain amount of compound for a given gas volume. It can be used for further experiments. Unless stated, NMR spectra were recorded at room temperature on a Bruker DPX 300 or a Bruker Avance 300 spectrometer. ^{1}H and $^{13}C\{^{1}H\}$ signals are referred to residual solvent signals, those of $^{31}P\{^{1}H\}$ to 85% H_3PO_4 and the ^{19}F NMR spectra to external $CFCl_3$. ^{1}H, ^{19}F and $^{31}P\{^{1}H\}$ signal assignments were confirmed by $^{1}H\{^{31}P\}$, $^{1}H\{^{19}F\}$, $^{19}F\{^{1}H\}$, $^{1}H,^{1}H$ COSY, $^{19}F,^{19}F$ COSY or $^{31}P,^{31}P$ COSY, $^{1}H,^{31}P$ HSQC NMR experiments. The determined coupling constant values of the higher order spectra were confirmed by *gNMR* software simulations. Infrared spectra were recorded inside a glovebox on a Bruker ALPHA II spectrometer equipped with an ATR-module (diamond). The Shimadzu GC-2014 gas chromatograph with a thermal conductivity detector and a Resteks ShinCarbon packed column ST 80/100 (2 m, 1/8" outer diameter, 2 mm inner diameter) was used for CO detection.

1.2 Reaction of PMVE with $[Rh(H)(PEt_3)_3]$ (1)

In a Young NMR tube $[Rh(H)(PEt_3)_3]$ (**1**) (40 mg, 0.087 mmol) was dissolved in tol-d_8 (0.4 mL). The tube was cooled to 77 K, degassed *in vacuo*, and pressurized with PMVE to 0.2 bar (36 mg, 0.218 mmol). The reaction was monitored by NMR spectroscopy at variable temperature from 233K to 298K. The formation of intermediate **2** and an isomer of it **2'** in a ratio of 1.7:1 was observed at 273 K. Additionally, the generation of *Z*-$(F_3CO)CF{=}CF(PFEt_3)$ at this temperature was identified. After warming up the NMR tube to 298 K, conversion of **2**, **2'** and *Z*-$(OCF_3)CF{=}CF(PFEt_3)$ into complex **3** and complex **4** in a ratio of 1.3:1, as well as

trifluoroethylene (C_2F_3H:**4** in a 0.4:1 ratio), Et_3PF_2 and 1,2,2,2-tetrafluoroethyl trifluoromethyl ether OCF_3CF_3CFH was observed. A solution of the complexes **3** and **4** was not stable at 298 K and a slow transformation into complex [Rh(CO)(CF(OCF_3)CF_3)(PEt_3)$_2$] (**5**) after 30 minutes was observed. Monitoring the reaction at 298 K at various times revealed the evolution of **3** into **4**, while **4** converts into **5**. When the reaction was completed after a day, organic products were separated from the organometallic product **5** by a trap-to-trap distillation under inert atmosphere.

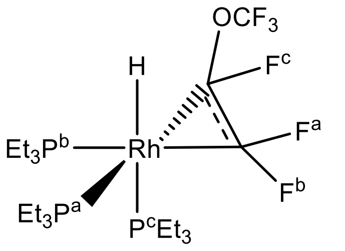

2

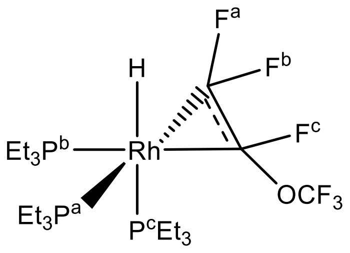

2'

Analytical data for **2 and 2':** ^{1}**H NMR** (600 MHz, Toluene-d_8) δ = 1.34-1.68 (m, P$CH_2$$CH_3$), 0.86-1.10 (m, P$CH_2$$CH_3$), -11.94 (dm, $^{2}J_{H,Pc}$= 152.8, dt in $^{1}H\{^{31}P\}$ NMR spectrum, $^{1}J_{H,Rh}$ =17.3 Hz, $^{3}J_{H,Fa/c}$ = 26.2, 1H, Rh*H* for **2'**), -12.12 (dm, $^{2}J_{H,\,Pc}$ =152.3, dd in $^{1}H\{^{31}P\}$ NMR spectrum, $^{3}J_{H,Fa}$ = 26.2, $^{1}J_{H,Rh}$ =17.3, 1H, RhH for **2**) ppm, part of the resonances corresponding for the phosphine ligands are overlapping. ^{31}**P{^{1}H} NMR** (273 K, 202 MHz, Toluene-d_8) δ 13.98-12.78 (dm, $^{1}J_{P,Rh}$ = 100.7, 1P, P^a/P^b), 11.16-9.39 (m, 1P, P^a/P^b), 4.85-2.64 (dm, $^{1}J_{P,Rh}$ = 91.3 Hz, 1P, P^c) ppm, the resonances corresponding to the phosphine ligands of **2** and **2'** are overlapping. ^{19}**F NMR** (253 K, 282.4 MHz, Toluene-d$_8$, isomer **2**) δ = -54.7 (t br, J = 8 Hz, 3F, OCF_3), -96.2 (dm, $^{2}J_{Fa,Fb}$ = 155 Hz, CF^bF^a), -112.5 (ddd br, $^{2}J_{Fb,Fa}$ = 154, $^{3}J_{Fb,Pb}$ =74, $^{3}J_{Fb,Pc}$ = 21 Hz, 1F, CF^bF^a), -129.8 (dt br, $^{3}J_{Fc,Pa}$ = 49, $^{3}J_{Fc,Pc} \approx$ $^{3}J_{Fc,Fa/b}$ = 29 Hz, 1F, CF^cOCF_3) ppm. ^{19}**F NMR** (253 K, 282.4 MHz, Toluene-d$_8$, isomer **2'**) δ = -54.8 (t br, $J_{F,F}$ = 8 Hz, 3F, OCF_3), -101.3 (dm, ddm br in $^{19}F\{^{1}H\}$ NMR spectrum, $^{2}J_{Fa,Fb}$ = 152, $^{3}J_{Fa,Pa}$ = 52, 1F, CF^bF^a), -108.2 (dm, dddm in $^{19}F\{^{1}H\}$ NMR spectrum, $^{2}J_{Fb,Fa}$ = 154, $^{3}J_{Fb,Pa}$ = 71, $^{3}J_{Fb,Pc}$ = 32 Hz, 1F, CF^bF^a), -119.4 (m, 1F, CF^cOCF_3) ppm.

The isomers have been assigned comparing the P,F and F,F, and Rh,F coupling constants with data for other rhodium olefin complexes.[5,6]

Analytical data for **3:** ^{1}**H NMR** (300.1 MHz, Toluene-d$_8$) δ = 5.81 (ddd, $^{2}J_{H,F}$ = 57.4, $^{3}J_{H,F}$ = 9.4 $^{3}J_{H,F}$ = 8.6 Hz, 1H, CFH, s in $^{1}H\{^{19}F\}$ NMR spectrum), 1.72-1.48 (m, P$CH_2$$CH_3$), 1.06-0.81 (m, P$CH_2$$CH_3$) ppm, the resonances corresponding to the phosphine ligands are overlapped with

the signals of other products. ^{31}**P{^{1}H}** NMR (121.5 MHz, Toluene-d_8) δ = 22.33 (dt br, $J_{P,Rh}$ = 138.3, $^{3}J_{P,F}$ = 24.4 Hz) ppm. ^{19}**F NMR** (282.4 MHz, Toluene-d8) δ = -58.4 (d br, $J_{F,F}$= 4 Hz, 3F, OCF_3), -92.4 (dm, $^{2}J_{F,F}$ = 295 Hz, 1F, C*F*F), -94.0 (dm, $^{2}J_{F,F}$ = 296 Hz, dt br at 213K, $^{2}J_{F,F}$ = 296, $^{3}J_{F,P}$ = 24 Hz ,1F, C*F*F), -134.9 (dm, $^{2}J_{F,H}$ = 57 Hz, 1F, CFH in ^{19}F{^{1}H} NMR spectrum appears as a br signal) ppm. **IR** (ATR, diamond): IR (cm^{-1}): $\tilde{\nu}$ = 1945 (CO).

Analytical data for **4:** ^{1}**H NMR** (300.1 MHz, Toluene-d_8): δ = 1.61 (qm, $^{3}J_{H,H}$ = 7.6 Hz, 12 H, PCH_2CH_3), 1.08 (dt, $^{3}J_{H,P}$ = 15.8, $^{3}J_{H,H}$ = 7.6 Hz, 18 H, PCH_2CH_3) ppm. ^{31}**P{^{1}H}** NMR (243 MHz, Toluene-d_8) δ = 25.31 (dd, $^{1}J_{P,Rh}$ = 127.8, $^{2}J_{P,F}$ = 26.1 Hz) ppm. ^{19}**F NMR** (282.4 MHz, Toluene-d_8) δ = -272.6 (dt, $^{1}J_{F,Rh}$ = 48, $^{2}J_{F,P}$ = 26 Hz) ppm.

The values are in accordance to the literature.[3]

Analytical data for **5:** ^{1}**H NMR** (300.1 MHz, C_6D_6) δ = 1.57-1.50 (m, q in ^{1}H{^{31}P}, $^{3}J_{H,H}$ = 7.43, 6H, PCH_2CH_3), 0.99 – 0.89 (m, t in ^{1}H{^{31}P}, $^{3}J_{H,H}$ = 7.43, 9H, PCH_2CH_3) ppm. ^{31}**P{^{1}H} NMR** (122 MHz, C_6D_6) δ = 24.36 (d, $^{1}J_{P,Rh}$ = 120.9 Hz) ppm. ^{19}**F NMR** (282.4 MHz, C_6D_6) δ = -53.7 (dq, $^{3}J_{F,F}$ = 10, $^{5}J_{F,F}$ = 1 Hz, 3F, CF_3), -81.5 (dq, $^{4}J_{F,F}$ = 3, $^{5}J_{F,F}$ = 1 Hz, 3F, OCF_3), -129.5 (qq, $^{3}J_{F,F}$ = 10, $^{4}J_{F,F}$ = 3 Hz, 1F, CF) ppm. **IR** (ATR, diamond): IR (cm^{-1}): $\tilde{\nu}$ = 1938 (CO).

Analytical data for **CF_2=CFH:** ^{1}**H NMR** (300.1 MHz, Toluene-d_8) δ = 5.63 (ddd, $^{2}J_{F,H}$ = 70.4, $^{3}J_{F,H}$ = 13.2, $^{3}J_{F,H}$ = 4.3 Hz, s in ^{1}H{^{19}F} NMR spectrum, 1H, CF*H*) ppm, ^{19}**F NMR** (282.4 MHz, Toluene-d_8) δ = -100.7 (ddd, $^{2}J_{F,F}$ = 84, $^{3}J_{F,F}$ =33, $^{3}J_{F,H}$ =13 Hz, dd in ^{19}F{^{1}H} NMR spectrum, 1F, CFF), -126.5 (ddd, $^{3}J_{F,F}$ = 118, $^{2}J_{F,F}$ = 83, $^{3}J_{F,H}$ = 4 Hz, dd in ^{19}F{^{1}H} NMR spectrum, 1F, CF*F*), -204.3 (ddd, $^{3}J_{F,F}$ = 118, $^{2}J_{F,H}$ = 70, $^{3}J_{F,F}$ = 33 Hz, dd in ^{19}F{^{1}H} NMR spectrum, 1F, C*F*H) ppm.

The values are in accordance to the literature.[7]

Analytical data for **CHF(OCF_3)(CF_3):** ^{1}**H NMR** (300.1 MHz, C_6D_6) δ = 4.96 (dq, $^{2}J_{H,F}$ = 53.1, $^{3}J_{H,F}$ = 2.8 Hz, s in ^{1}H{^{19}F}, 1H, C*H*) ppm. ^{19}**F NMR** (282.4 MHz, C_6D_6) δ = -60.0 (d, $^{4}J_{F,F}$ = 4 Hz, 3F, OCF_3), -84.1 (dd, $^{3}J_{F,F}$ = 6, $^{3}J_{F,H}$ = 3 Hz, d in ^{19}F{^{1}H} NMR spectrum, 3F, CF_3), -146.0 (dqq, $^{2}J_{F,H}$ = 53, $J_{F,F}$ = 6, $^{4}J_{F,F}$ = 4 Hz, qq in ^{19}F{^{1}H} NMR spectrum, 1F, C*F*) ppm.

The values are in accordance to the literature.[8]

1.3 Independent reaction of PMVE with [Rh(F)(CO)(PEt_3)$_2$] (4)

In a Young NMR tube [Rh(F)(CO)(PEt_3)$_2$] (**4**) (40 mg, 0.104 mmol) was dissolved in C_6D_6 (0.4 mL). The reaction mixture was cooled to 77 K, degassed *in vacuo*, and pressurized with PMVE to 0.2 bar. NMR studies show at 298K the formation of **5**.

1.4 Reaction of PMVE with triethylphosphine PEt$_3$

In a in a PFA inliner tube triethylphosphine (25 µl, 0.169 mmol) was dissolved in C_6D_6 (0.4 mL). The tube was cooled to 77 K, degassed *in vacuo*, and pressurized with PMVE to 0.2 bar (36 mg, 0.220 mmol). The tube was sealed by melting, right after the addition of the PMVE gas. NMR spectroscopy after 10 minutes shows at 298K the formation of fluorophosphoranes *Z/E*-(OCF_3)CF=CF($PFEt_3$) in a 10:1 ratio. At 298K, after 30 minutes, the colour of the solution changed from yellow to red. Based on the $^{31}P\{^1H\}$ NMR spectrum, a full conversion into Et_3PF_2 was observed. In the ^{19}F NMR spectrum in addition to the formation of Et_3PF_2 some unidentified decomposition products, presumably due to the further reactivity of the C_2F_4, were observed. The $^{13}C\{^1H\}$ NMR spectrum reveals the formation of CO. Using a gas tight syringe, 250 µL of the gas phase was injected into the gas chromatograph (GC) the CO with a retention time of 2.8 min^{-1} could be detected at an oven temperature of 40°C.

Analytical data for ***Z/E*** **-($F^d{}_3CO$)CF^a=CF^b(PF^cEt_3):**

^{1}H NMR (300.1 MHz, C_6D_6, *Z* isomer) δ = 1.82 (dq, $^2J_{H,P}$ = 16.6, $^3J_{H,H}$ = 7.8 Hz, 6H, PCH_2CH$_3$), 1.03 (dt, $^3J_{H,P}$ = 23.4, $^3J_{H,H}$ = 7.8 Hz, 9H, PCH$_2$$CH_3$) ppm, the resonances corresponding to the *E* isomer are overlapped with these signals. **$^{31}P\{^1H\}$ NMR** (121.5 MHz, C_6D_6) δ = -63.57 (dt br, $^1J_{P,Fc}$ = 598.8, $^2J_{P,F}$ = 2.4 Hz, For the *Z* isomer), -63.76 (ddd br, $^1J_{P,Fc}$ = 592.2, $^2J_{P,Fa}$ = 15.4, $^2J_{P,Fb}$ = 1.4 Hz, For the *E* isomer) ppm. **^{19}F NMR** (282.4 MHz, C_6D_6, *Z* isomer) δ = -19.6 (dm, $^1J_{Fc,P}$ = 599 Hz, dd br in $^{19}F\{^1H\}$ NMR spectrum $^1J_{Fc,P}$ = 599, $^3J_{Fc,Fb}$ = 14 Hz, F^c), -59.9 (dd br, $^5J_{Fd,Fb}$ = 6, $^4J_{Fd,Fa}$ = 4 Hz, 3F, OC$F^d{}_3$), -118.9 (d br, $^3J_{Fa,Fb}$ = 116 Hz dm in $^{19}F\{^1H\}$ $^3J_{Fa,Fb}$= 116, $^4J_{Fa,Fd}$ = 4 Hz, 1F, F^a), -156.3 (dm, $^3J_{Fb,Fa}$ = 115 Hz ddqd in $^{19}F\{^1H\}$ NMR spectrum, $^3J_{Fb,Fa}$ = 115, $^3J_{Fb,Fc}$ = 14, $^4J_{Fb,Fd}$ = 6, $^2J_{Fb,P}$ = 2 Hz, 1F, F^b) ppm. **^{19}F NMR** (282.4 MHz, C_6D_6, *E* isomer) δ = -22.3 (dm, $^1J_{Fc,P}$ = 592, ddd in $^{19}F\{^1H\}$ NMR spectrum, $^1J_{Fc,P}$ = 592, $^3J_{Fc,Fb}$ = 12, $^4J_{Fc,Fa}$ = 11 Hz, 1F, F^c), -58.9 (d br, $^4J_{Fd,Fa}$= 6 Hz, 3F, OC$F^d{}_3$), -105.4 (m, dm in $^{19}F\{^1H\}$ NMR spectrum, $^3J_{Fa,Fb}$ = 25, 1F, F^a), -152.5 (ddm, ddd in $^{19}F\{^1H\}$ NMR spectrum, $^3J_{Fb,Fa}$ = 25, $^3J_{Fb,Fc}$ = 12, $^2J_{Fb,P}$ = 2 Hz, 1F, F^b) ppm.

The values are in accordancee to the literature for other olefins.[9-11]

Selected analytical data for **Et_3PF_2**: **^{1}H NMR** (300.1 MHz, C_6D_6) δ = 1.83 (dtq, $^2J_{H,P}$ = 15.8, $^3J_{H,F}$ = 10.9, $^2J_{H,H}$ = 7.8 Hz, 6H, P*CH2*CH3), 1.05 (dtt, $^3J_{H,P}$ = 22.7, $^3J_{H,H}$ = 7.8, $^4J_{H,F}$ = 1.1 Hz, 9H, PCH2*CH3*) ppm. **^{31}P{^{1}H} NMR** (121.5 MHz, C_6D_6) δ = -11.00 (t, $^1J_{P,F}$ = 584.9 Hz) ppm. **^{19}F NMR** (282.4 MHz, C_6D_6) δ = -39.5 (ddm, $^1J_{F,P}$ = 585, $^3J_{F,H}$ = 11 Hz) ppm.

The values are in accordance to the literature. [12]

Analytical data for **CO**:

^{13}C{^{1}H} NMR (75.5 MHz, C_6D_6) δ = -184.1 (s, CO) ppm.

This value are in accordance to the literature.[13]

1.5 Independent reaction of fluorophosgene with triethylphosphine PEt_3

In a Young NMR tube triethylphosphine (25 µl, 0.169 mmol) was dissolved in C_6D_6 (0.4 mL). The tube was cooled to 77 K, degassed *in vacuo*, and pressurized with COF_2 to 0.2 bar. NMR spectroscopy after 10 minutes shows at 273K the formation Et_3PF_2.

1.6 Reaction of 1 equivalent of the PMVE with [Rh(H)(PEt_3)$_3$] (1)

In a Young NMR tube [Rh(H)(PEt_3)$_3$] (**1**) (50 mg, 0.109 mmol) was dissolved in C_6D_6 (0.4 mL). The reaction mixture was cooled to 77 K, degassed *in vacuo*, and pressurized with PMVE to 0.1 bar (18 mg, 0.109 mmol). NMR studies reveal the formation of **7** at 298K, as well as fluorophosphoranes F_2PEt_3, and phosphine oxide.

1.7 Independent reaction of trifluoroethylene with [Rh(F)(PEt_3)$_3$] (6)

In a Young NMR tube [Rh(F)(PEt_3)$_3$] (**4**) (22 mg, 0.046 mmol) was dissolved in C_6D_6 (0.4 mL). The reaction mixture was cooled to 77 K, degassed *in vacuo*, and pressurized with trifluoroethylene to 0.2 bar (14 mg, 0.171 mmol). NMR studies show at 298K the formation of **7**, as well as free phosphine PEt_3.

Analytical data for **7**: **^{1}H NMR** (300.1 MHz, C_6D_6): δ = 5.50 (ddd, $^2J_{H,Fc}$ = 73.3 Hz, $^3J_{H,Pb}$ = 8.8, $^3J_{H,F}$ = 2.7 Hz, dd in the ^{1}H{^{31}P} NMR spectrum, CF*H*), 1.66 (m, $^3J_{H,H}$ = 7.59 Hz, 6H, P*CH2*CH3), 1.25 (m, quint br, $^2J_{H,P} \approx {}^3J_{H,H}$ = 7.7 Hz, 6H, PC*H2*CH3), 1.08 (m, t in ^{1}H{^{31}P} NMR spectrum, $^3J_{H,H}$ = 7.6 Hz, 9H, PCH2*CH3*), 0.96 (m, dt in ^{1}H{^{31}P} NMR spectrum, $^3J_{H,F}$ = 11.4, $^3J_{H,H}$ = 7.7 Hz, 9H, PCH2C*H3*) ppm. **^{31}P{^{1}H} NMR** (121.5 MHz, C_6D_6): δ = 32.3 (ddt br, $^2J_{Pa,Pb}$= 368.2 Hz, $^1J_{Pa,Rh}$ = 138.9 Hz, $^3J_{Pa,Fb} \approx {}^3J_{Pa,Fc}$= 47.9 Hz, 1P, P^a), 25.9 (dddd, $^2J_{Pb,Pa}$ = 368.2 Hz,

$^{1}J_{Pb,Rh}$ = 133.3 Hz, $^{3}J_{Pb,Fa}$ = 44.2 Hz, $^{2}J_{P,F}$ = 14.8 Hz, 1P, P^{b}) ppm. **^{19}F NMR** (282.4 MHz, C_6D_6): δ = -89.4 (dddd, $^{2}J_{Fa,Fb}$ = 109, $^{3}J_{Fa,Pb}$ = 46, $^{3}J_{Fa,Fc}$ = 33, $^{2}J_{Fa,Rh}$ =13 Hz, 1F, C*F^{a}*F), -90.8 (dddd, $^{2}J_{Fb,Fa}$ = 109 Hz, $^{3}J_{Fb,Fc}$ = 69 Hz, $^{3}J_{Fb,Pa}$ = 44, $^{2}J_{Fb,Rh}$ = 4 Hz, CF*F^{b}*), -194.9 (m, dddm in ^{19}F{^{1}H} NMR spectrum, $^{3}J_{Fc,Fb}$ = 69, $^{3}J_{Fc,Pa}$ = 48, $^{3}J_{Fc,Fa}$ = 32, 1F, C*F^{c}*H), -218.3 (m, Rh*F*) ppm. NMR assignments of the CF_2CFH moiety are consistent with previously reported η^2-coordinated trifluoroethylene at a nickel complex.[17]

1.8 Reaction of PMVE with [Rh(F)(PEt$_3$)$_3$] (6)

In a Young NMR tube [Rh(F)(PEt_3)$_3$] (**6**) (40 mg, 0.084 mmol) was dissolved in C_6D_6 (0.4 mL). The solution was cooled to 77 K, degassed *in vacuo*, and pressurized with PMVE to 0.2 bar (36 mg, 0.218 mmol). NMR studies show at 298K the formation of **8** and **4** and **5**, as well as fluorophosphoranes F_2PEt_3, and traces of $[Rh(PEt_3)_4]^+$. At the beginning of the reaction, *Z*-(OCF_3)CF=CF($PFEt_3$) was formed with no indications for the formation of **4** or **5**. However, after 3 hours, as the fluorophosphorane decomposed, a mixture of complexes **8** and **4** in a 2.4:1 ratio, and the Et_3PF_2 were observed. Additionally, the formation of complex **5** slowly took place. A similar reaction was conducted in non-dried C_6D_6, and only **8** along with phosphine oxide Et_3P=O were formed.

Analytical data for **8**: **^{1}H NMR** (500.1 MHz, C_6D_6) δ = 1.66 -1.34 (m, overlapped with signals of other Et groups, 12H, PC*H$_2$*CH$_3$), 1.13 - 0.91 (m, overlapped with signals of other Et groups, 18H, PCH$_2$*CH$_3$*) ppm. **^{31}P{^{1}H} NMR** (242 MHz, C_6D_6) δ = 34.09 (ddtd, $^{2}J_{P,Pa}$ = 366.7, $^{1}J_{P,Rh}$ = 133.4, $^{3}J_{P,Fa} \approx {}^{3}J_{P,Fc}$ = 45.0, $^{2}J_{P,Fd}$ = 17.3 Hz, 1P, P^{b}), 30.52 (dddd, $^{2}J_{P,Pb}$ = 366.7, $^{1}J_{P,Rh}$ = 134.1, $^{3}J_{P,Fb}$ = 45.3, $^{2}J_{P,Fd}$ = 18.6 Hz, 1P, P^{a}) ppm, the spectrum is of higher order an coupling constants have been simulated. **^{19}F NMR** (282.4 MHz, C_6D_6) δ = -57.1 (t br, $^{4}J_{F,Fb} \approx {}^{5}J_{F,Fc}$ = 6 Hz, 3F, OCF_3), -96.8 (dmq, $^{2}J_{Fb,Fa}$ = 109, $^{3}J_{Fb,Pa}$ = 45, $^{5}J_{Fb,F}$ = 6 Hz, 1F, C*F^{b}*F^{a}), -98.2 (dddd, $^{2}J_{Fa,Fb}$ = 109, $^{3}J_{Fa,Pb}$ = 46, $^{3}J_{Fa,Fd}$ = 18, $^{2}J_{F,Rh}$ = 12 Hz, 1F, CF^{b}*F^{a}*), -116.1 (m, $^{4}J_{Fc,F}$ = 6 Hz, 1F, C*F^{c}*OCF$_3$), -206.35 (m, Rh*F^{d}*) ppm. The correlation of the olefinic fluorine atoms and the fluoride ligand was confirmed by ^{19}F,^{19}F COSY NMR spectrum.

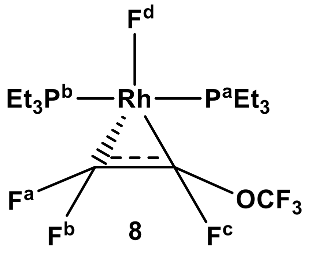

8

1.9 NMR Spectra

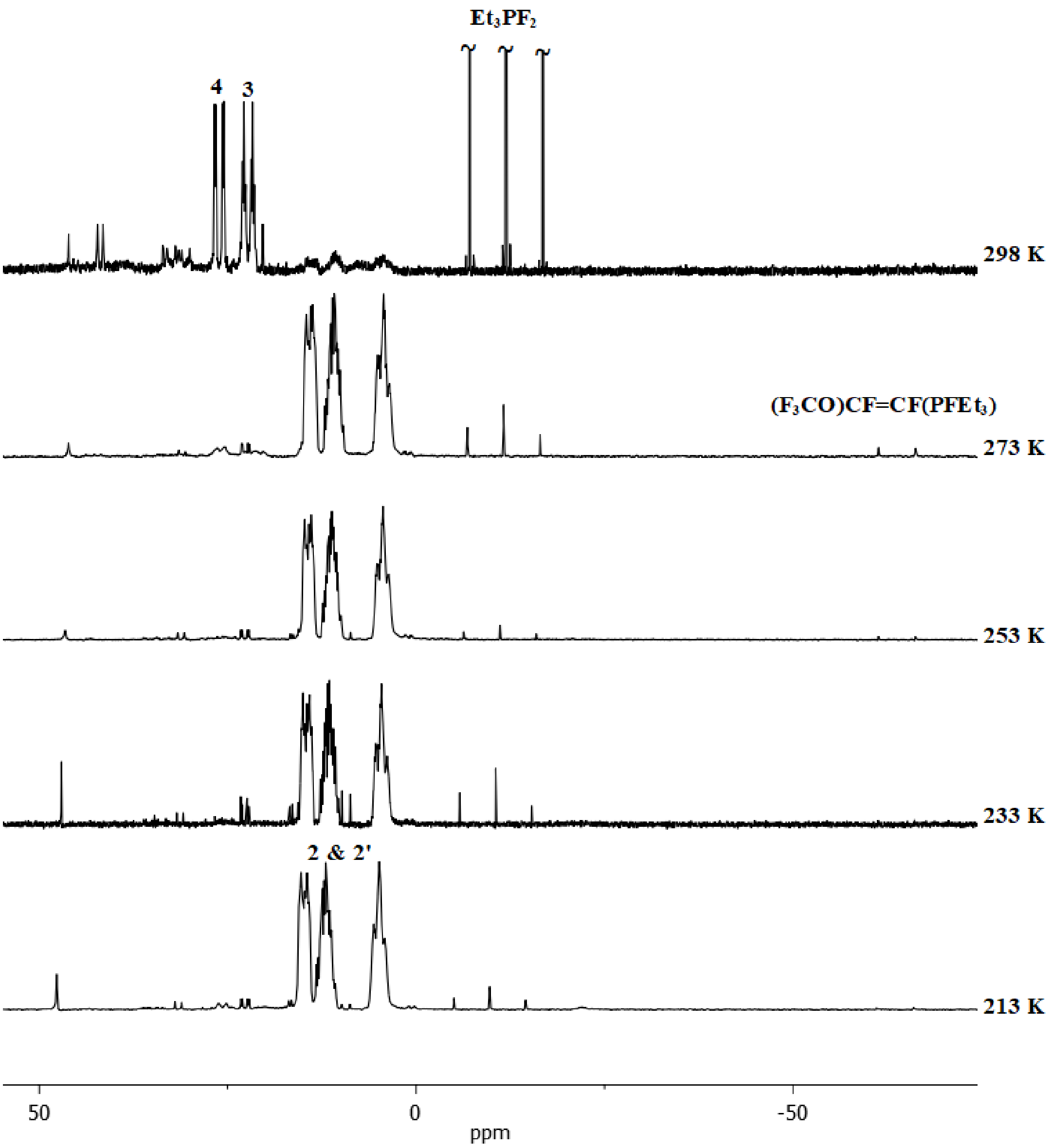

S1. $^{31}P\{^{1}H\}$ NMR spectrum of the reaction of complex **1** with excess of perfluoro (methyl vinyl ether) in toluene-d_8 at variable temperature showing the evolution of **2**, **2'** and $(F_3CO)CF{=}CF(PFEt_3)$ to complex **3**, **4** and Et_3PF_2.

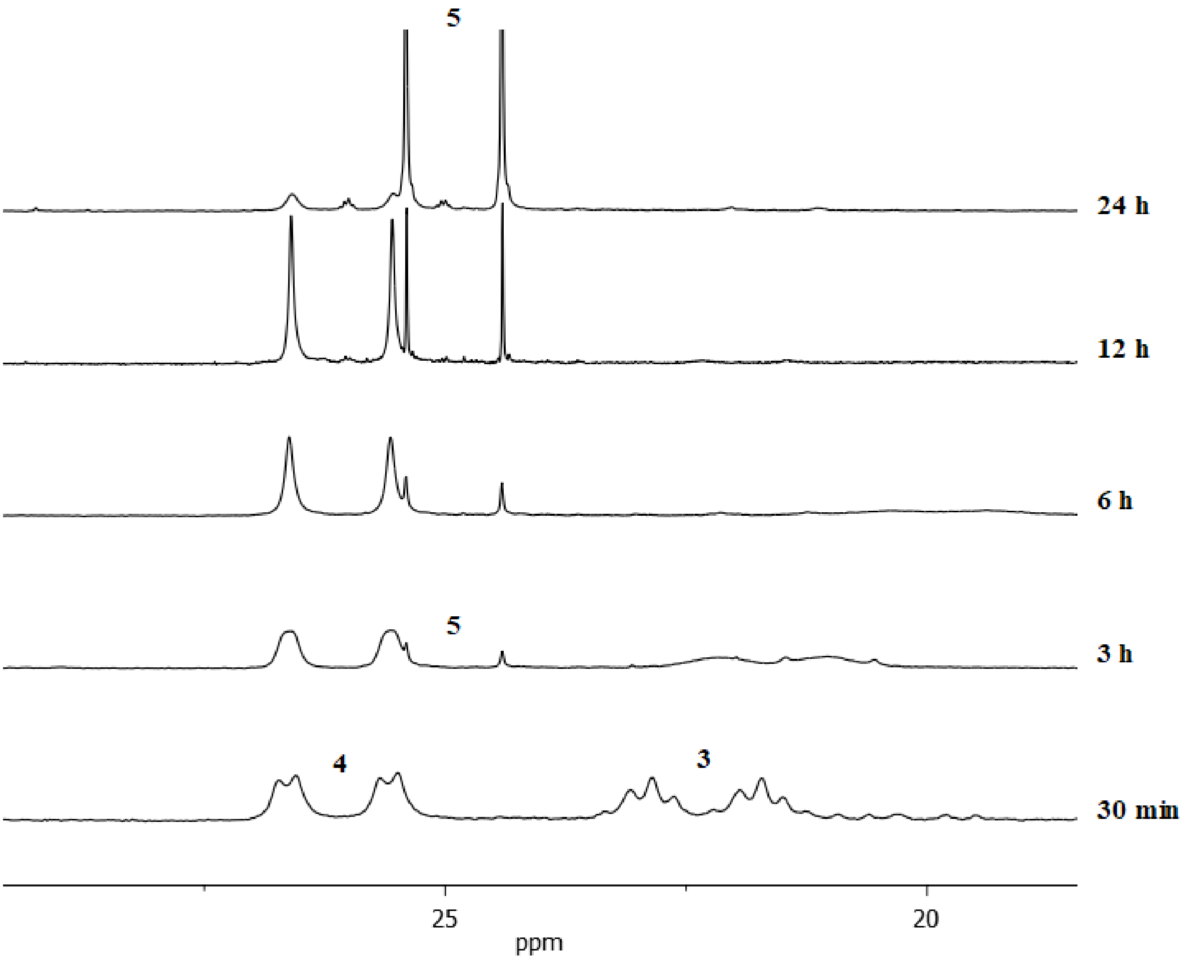

S2. $^{31}P\{^{1}H\}$ NMR spectrum of the reaction of complex **1** with excess of perfluoro (methyl vinyl ether) in C_6D_6 at room temperature at different time showing the evolution complex **3** to **4** and complex **4** to **5**.

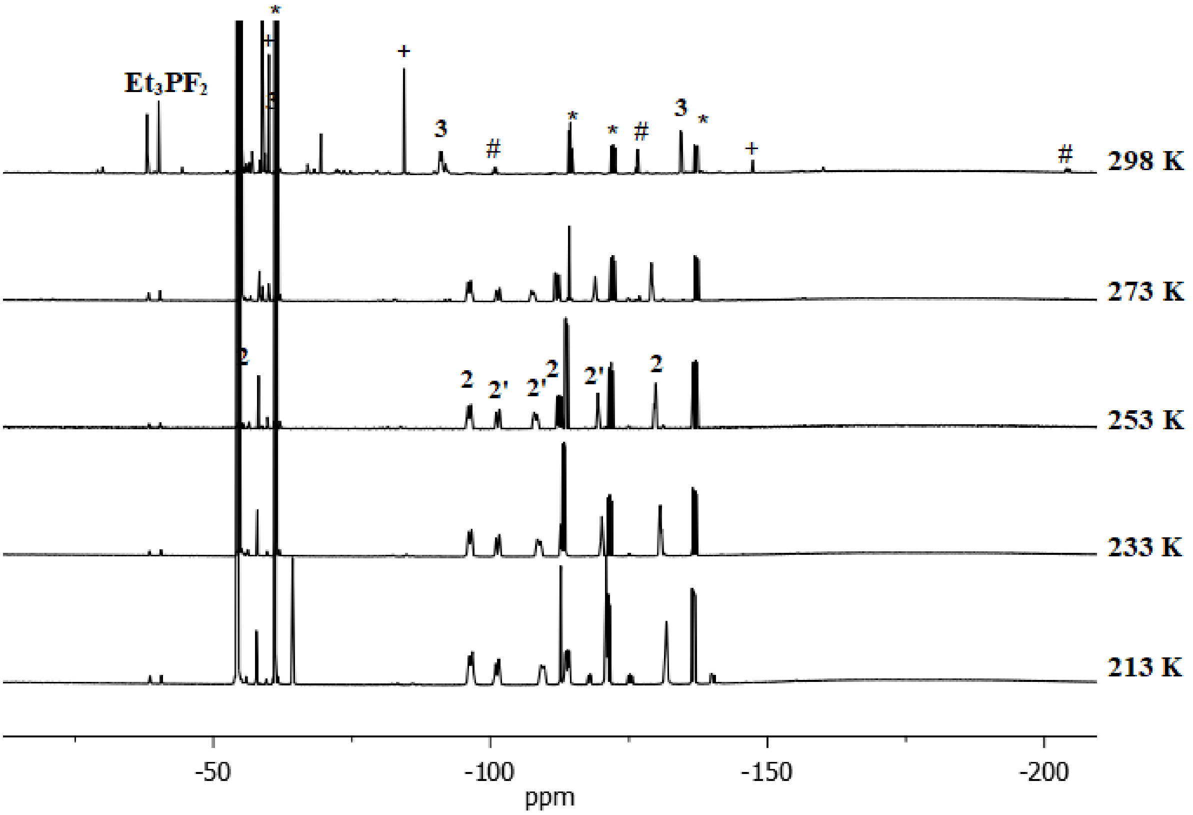

S3. ^{19}F NMR spectrum of the reaction of complex **1** with excess of perfluoro (methyl vinyl ether) in toluene-d_8 at variable temperature showing the evolution of **2**, **2'** and $(F_3CO)CF{=}CF(PFEt_3)$ to complex **3**, CF_2CFH (#), and OCF_3CF_3CFH (+), and Et_3PF_2. * = *PMVE.*

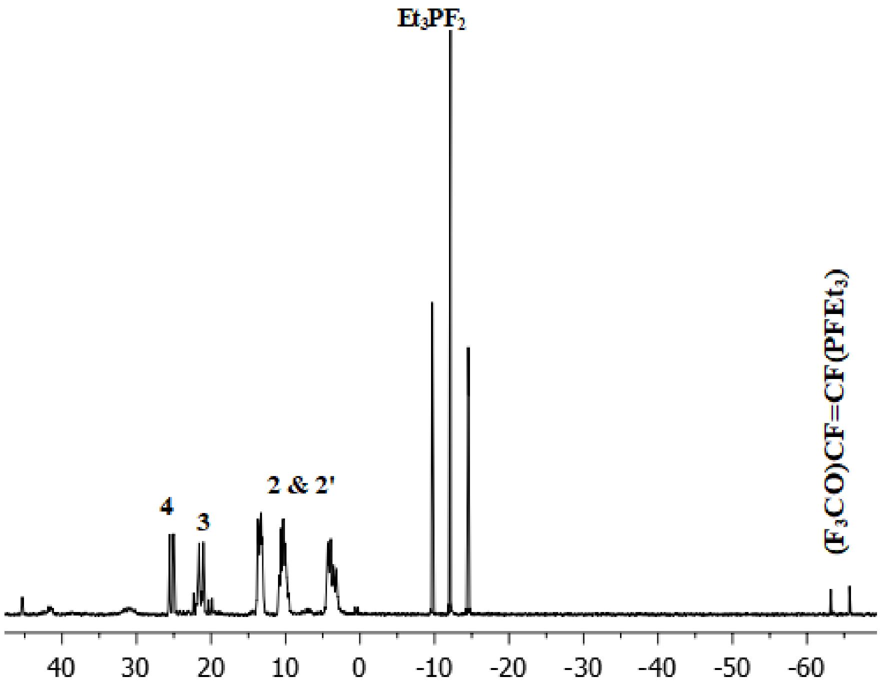

S4. ^{31}P{^{1}H} NMR spectrum (202 MHz) of the reaction of complex **1** with excess of perfluoro (methyl vinyl ether) in toluene-d_8 at 273 K.

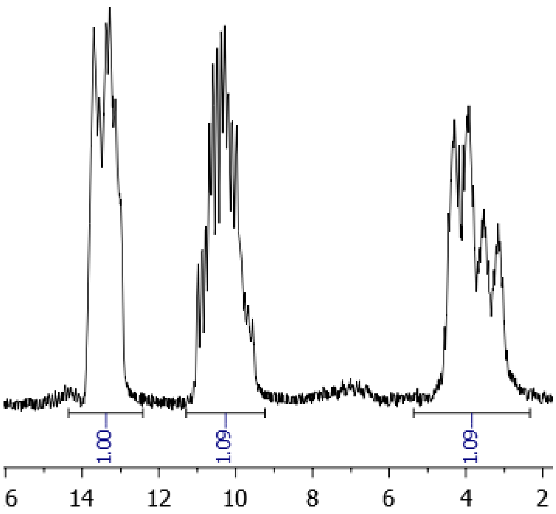

S5. Section of the $^{31}P\{^{1}H\}$ NMR spectrum (202 MHz) of the reaction of complex **1** with excess of perfluoro (methyl vinyl ether) in toluene-d_8 at 273 K showing the formation of **2** and **2'**.

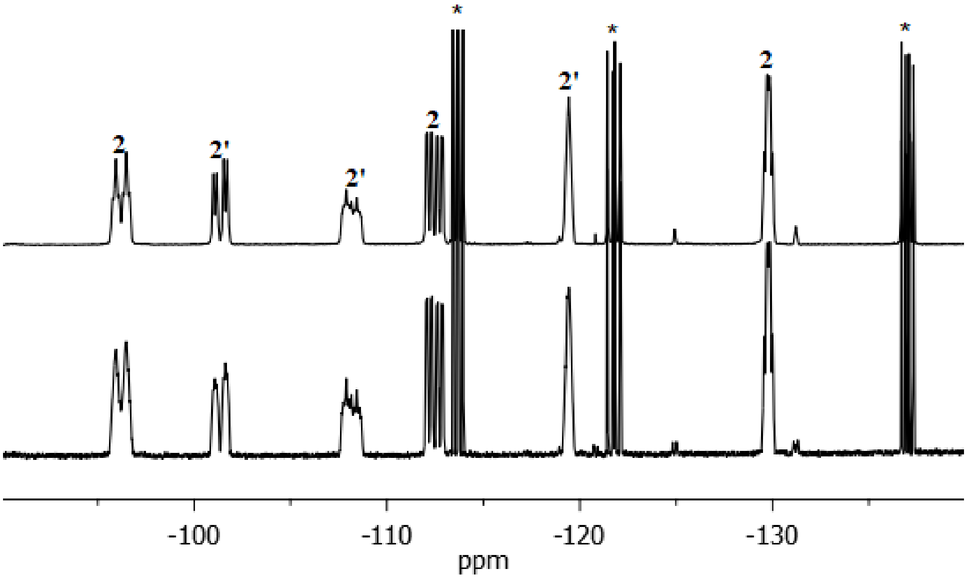

S6. Section of the ^{19}F NMR spectrum (below) and $^{19}F\{^{1}H\}$ NMR spectrum (top) of the reaction of complex **1** with excess of perfluoro (methyl vinyl ether) in toluene-d_8 at 273 K showing the formation of **2** and **2'**. * = *PMVE.*

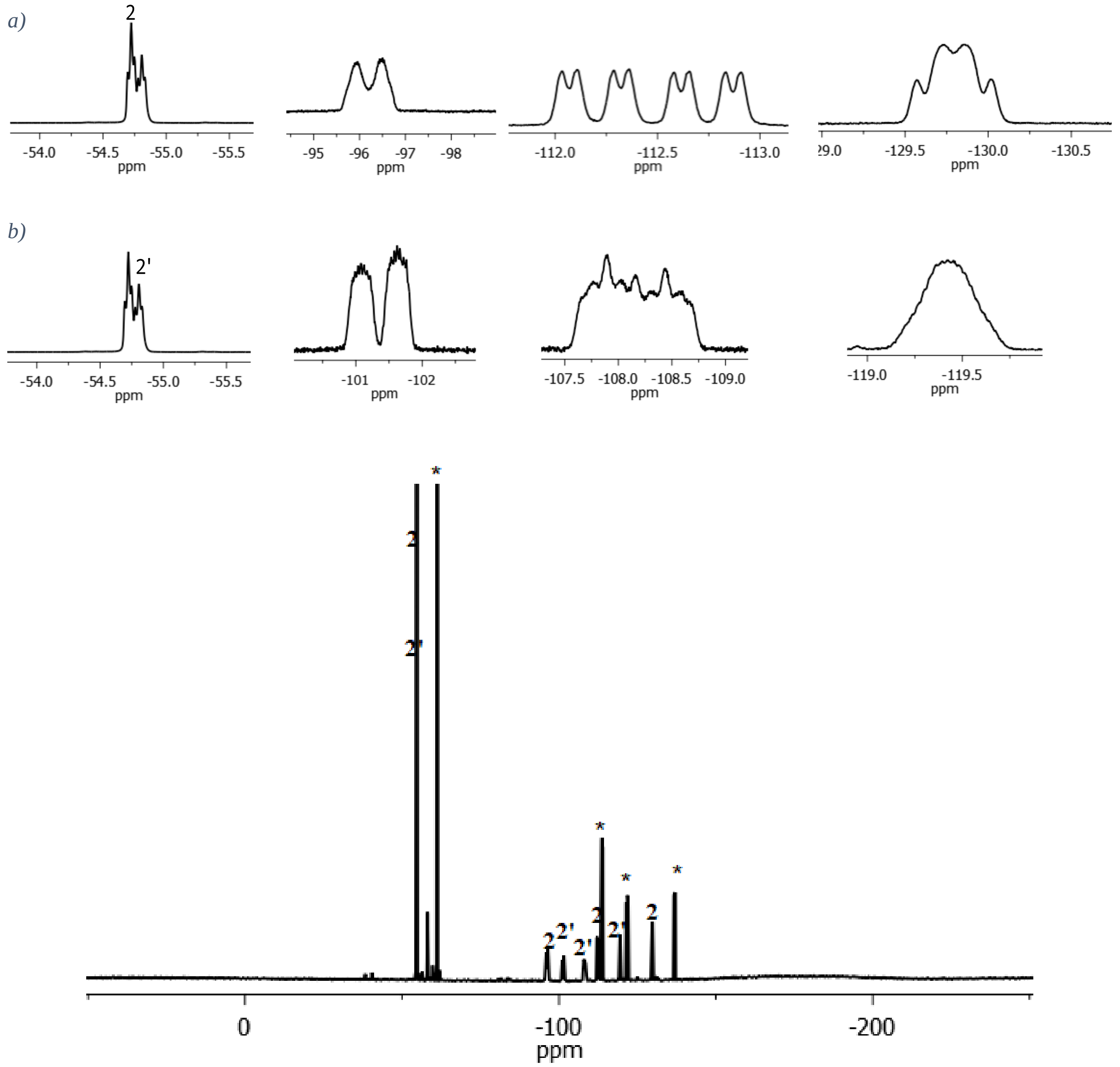

S7. ^{19}F NMR NMR spectrum of the reaction of complex **1** with excess of perfluoro (methyl vinyl ether) in toluene-d_8 at 273 K showing the formation of **2 (**zoomed in section a) and **2' (**zoomed in section b). * = *PMVE.*

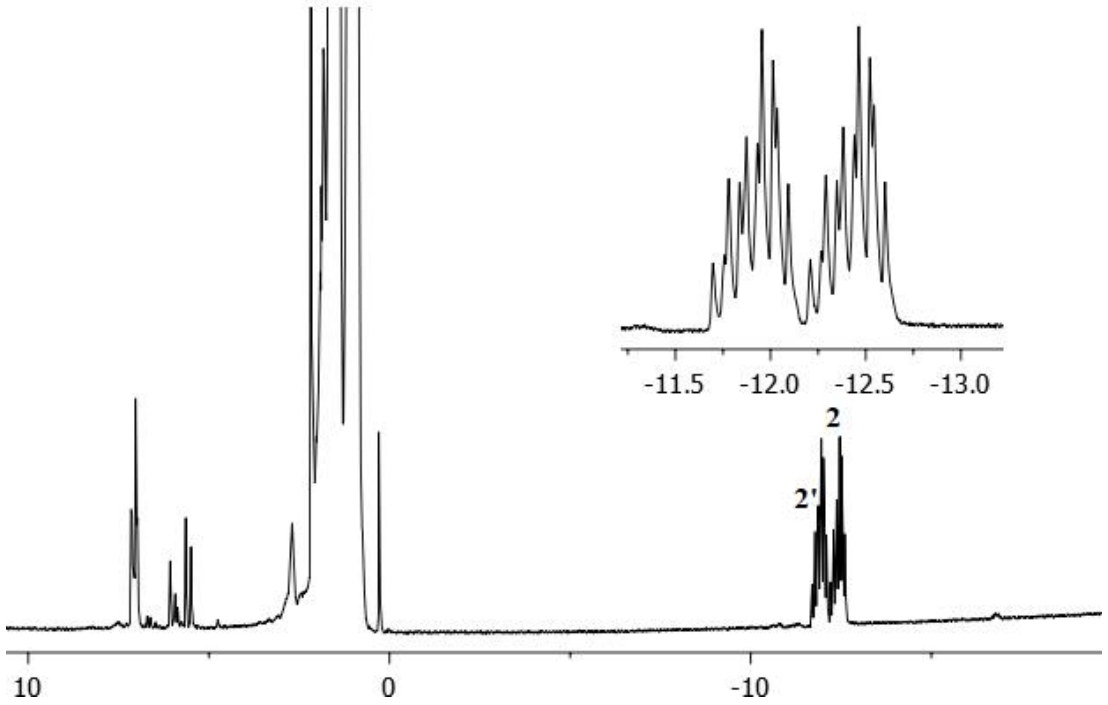

S8. ^{1}H NMR spectrum of the reaction of complex **1** with excess of perfluoro (methyl vinyl ether) in toluene-d_8 at 273 K showing the formation of **2** and **2'**: Zoomed in for the Metal-*H* region.

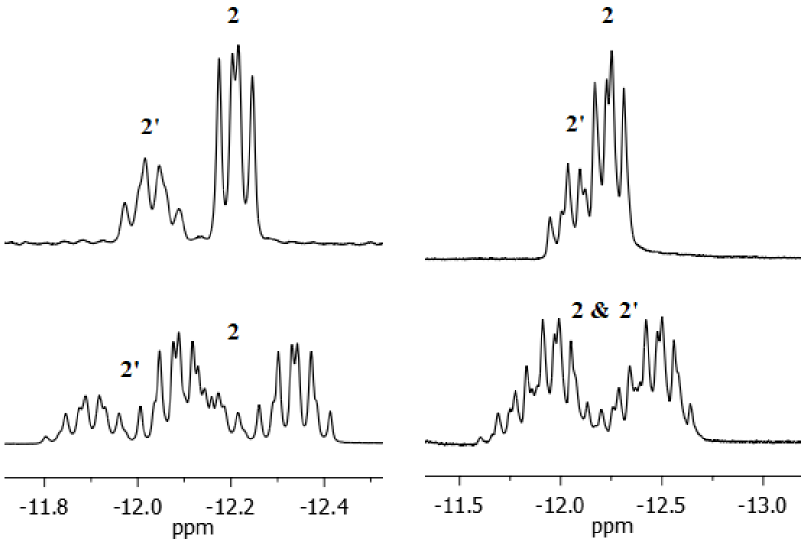

S9. Section of the ^{1}H NMR spectrum (bottom) and $^{1}H\{^{31}P\}$ NMR spectrum (top) of the reaction of complex **1** with excess of perfluoro (methyl vinyl ether) in toluene-d_8 at 273 K showing the formation of **2** and **2'** at 300 MHz (right) and 600 MHz (left) NMR spectrometers.

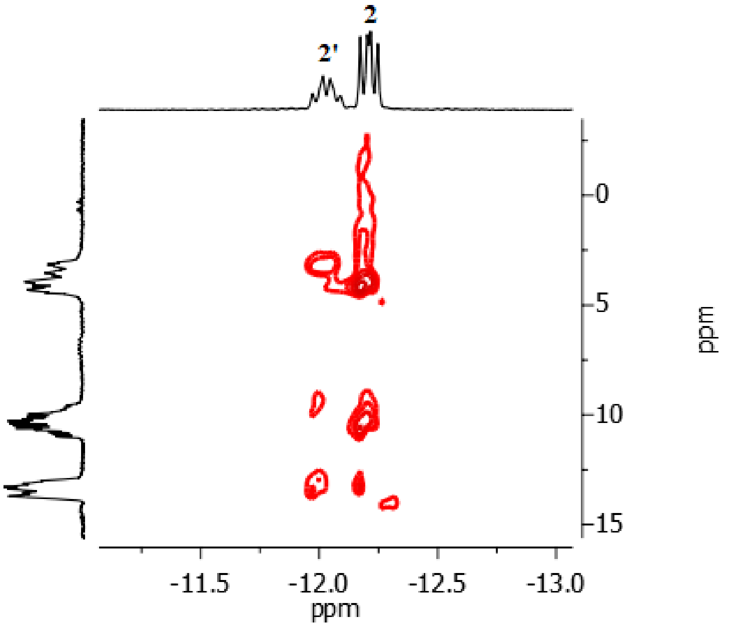

S10. Section of the ^{1}H-^{31}P HSQC NMR spectrum (600 MHz) of the reaction of complex **1** with excess of perfluoro (methyl vinyl ether) in toluene-d_8 at 273 K showing the formation of **2** and **2'**. *$^{1}H\{^{31}P\}$ (X axis) and $^{31}P\{^{1}H\}$ (Y axis) are externally projected for clarity.*

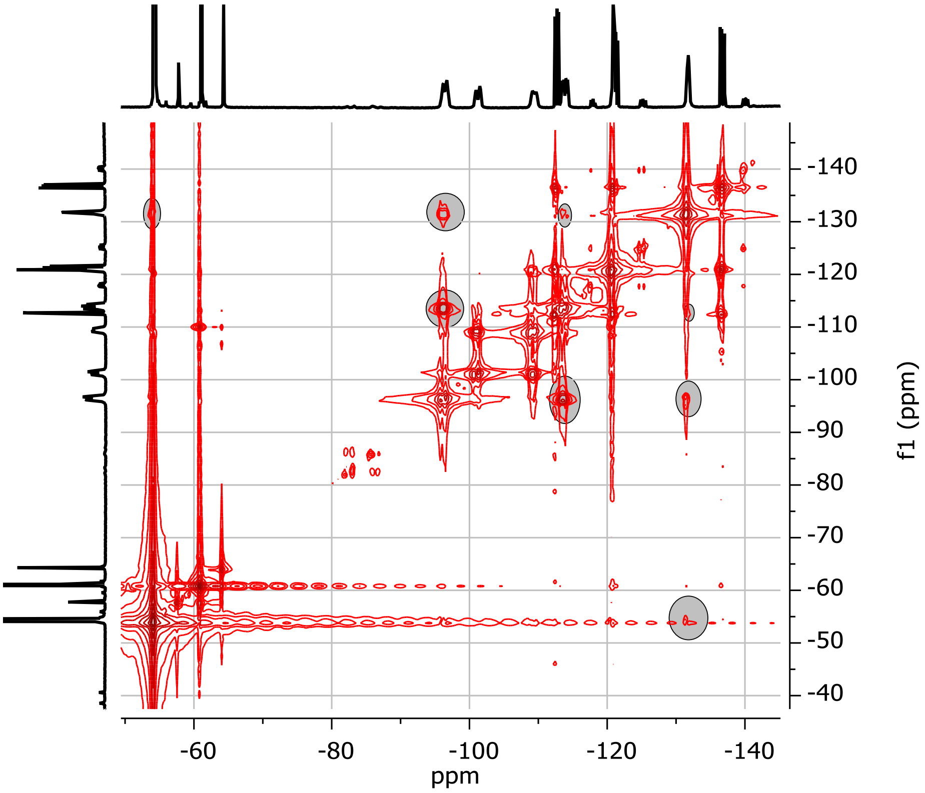

S11. ^{19}F-^{19}F COSY NMR spectrum of the reaction of complex **1** with excess of perfluoro (methyl vinyl ether) in toluene-d_8 at 273 K showing the formation of **2** and **2'**.

a)

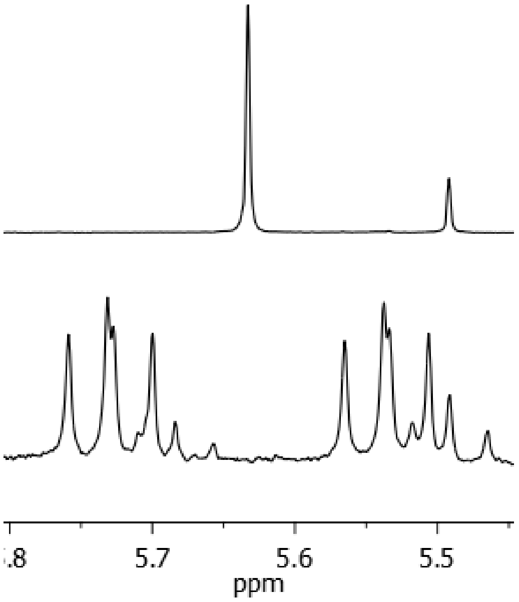

S12. Section of the ^{1}H NMR spectrum (bottom) and $^{1}H\{^{19}F\}$ NMR spectrum (top) of the reaction of complex **1** with excess of perfluoro (methyl vinyl ether) in toluene-d_8 showing the formation of **3**. The overlapping peak could belong to the probable isomer of **3** *trans*-[Rh(CF(OCF_3)CF_2H)(CO)(PEt_3)$_2$].

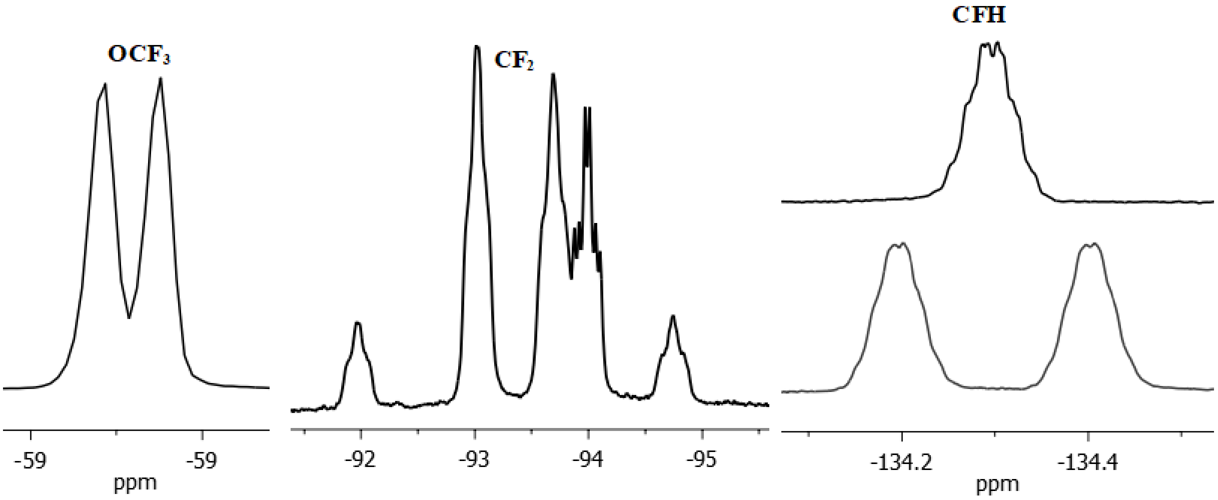

S13. Section of the ^{19}F NMR spectrum (bottom) and $^{19}F\{^{1}H\}$ NMR spectrum (top for the CFH group) of the reaction of complex **1** with excess of perfluoro (methyl vinyl ether) in toluene-d_8 showing the formation of **3**. The overlapping peak in the middle of the signal for the CF_2 group could belong to the probable isomer of **3** *trans*-[Rh(CF(OCF_3)CF_2H)(CO)(PEt_3)$_2$].

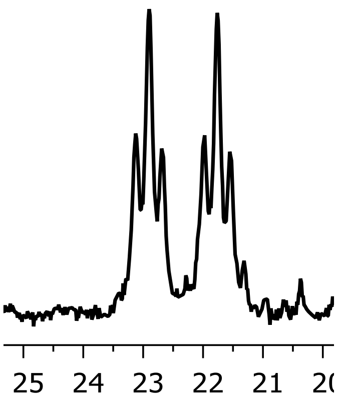

S14. Section of the $^{31}P\{^{1}H\}$ NMR spectrum of the reaction of complex **1** with excess of perfluoro (methyl vinyl ether) in toluene-d_8 showing the formation of **3**.

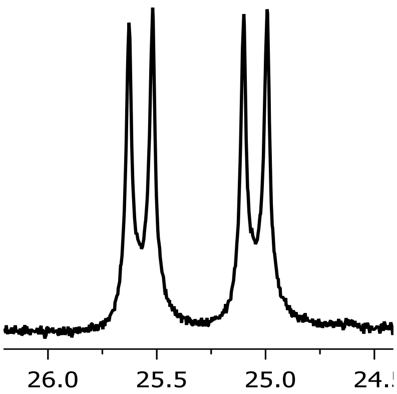

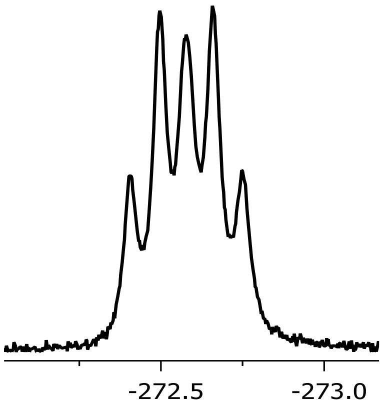

S15. Section of the $^{31}P\{^{1}H\}$ NMR spectrum (left) and the ^{19}F NMR spectrum (right) of the reaction of complex **1** with excess of perfluoro (methyl vinyl ether) in toluene-d_8 showing the formation of **4**.

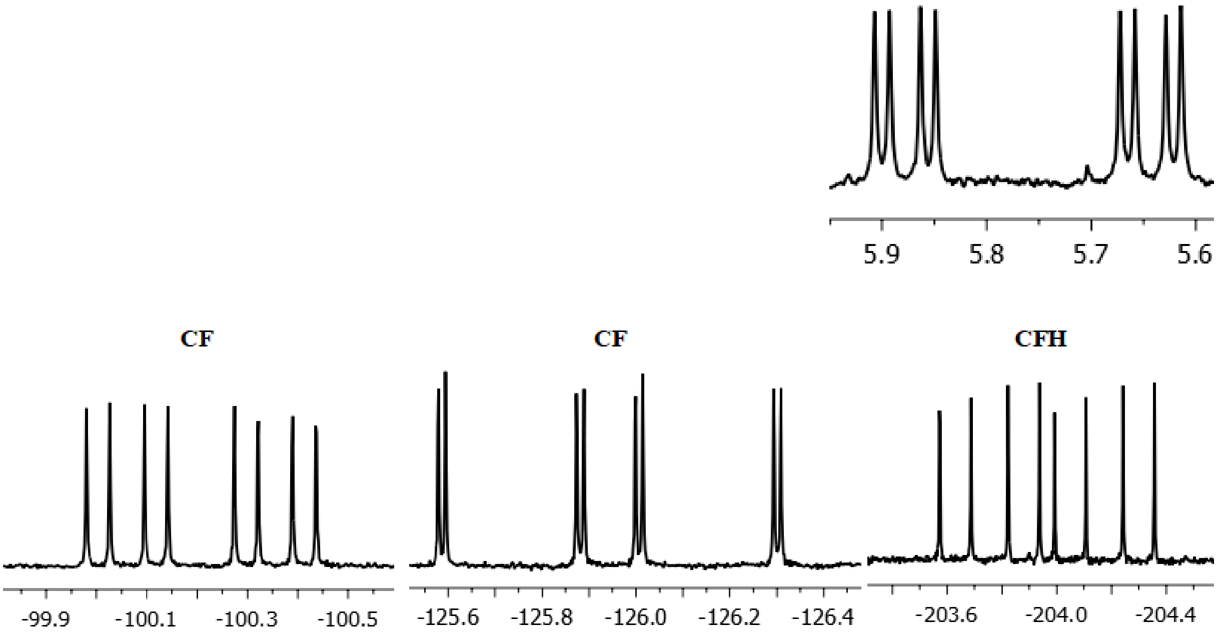

S16. Section of the ^{19}F NMR spectrum (bottom) and ^{1}H NMR spectrum (top) of the reaction of complex **1** with excess of perfluoro (methyl vinyl ether) in C_6D_6 showing the formation of trifluoroethylene CF_2=CFH.

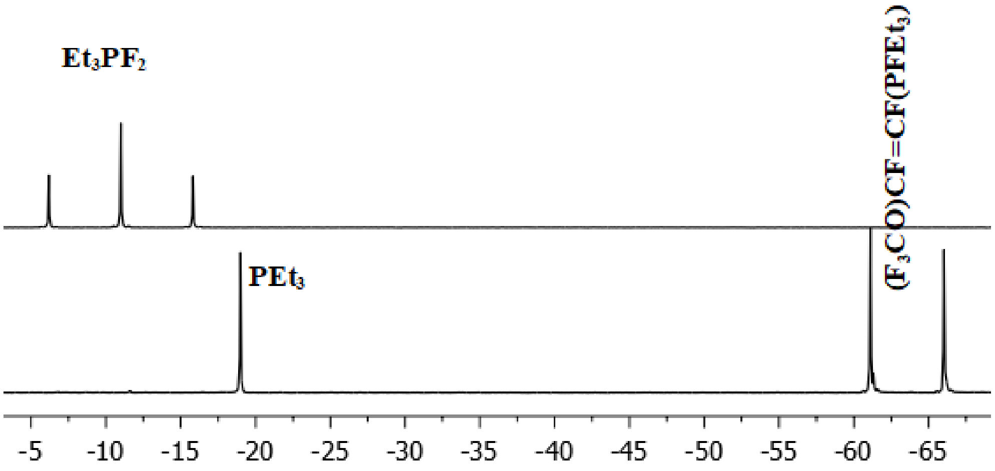

S17. $^{31}P\{^{1}H\}$ NMR spectrum of the reaction of PEt_3 with excess of perfluoro (methyl vinyl ether) in C_6D_6 showing the formation of $(F_3CO)CF=CF(PFEt_3)$ after 10 minutes (bottom) and Et_3PF_2 after 30 minutes (top).

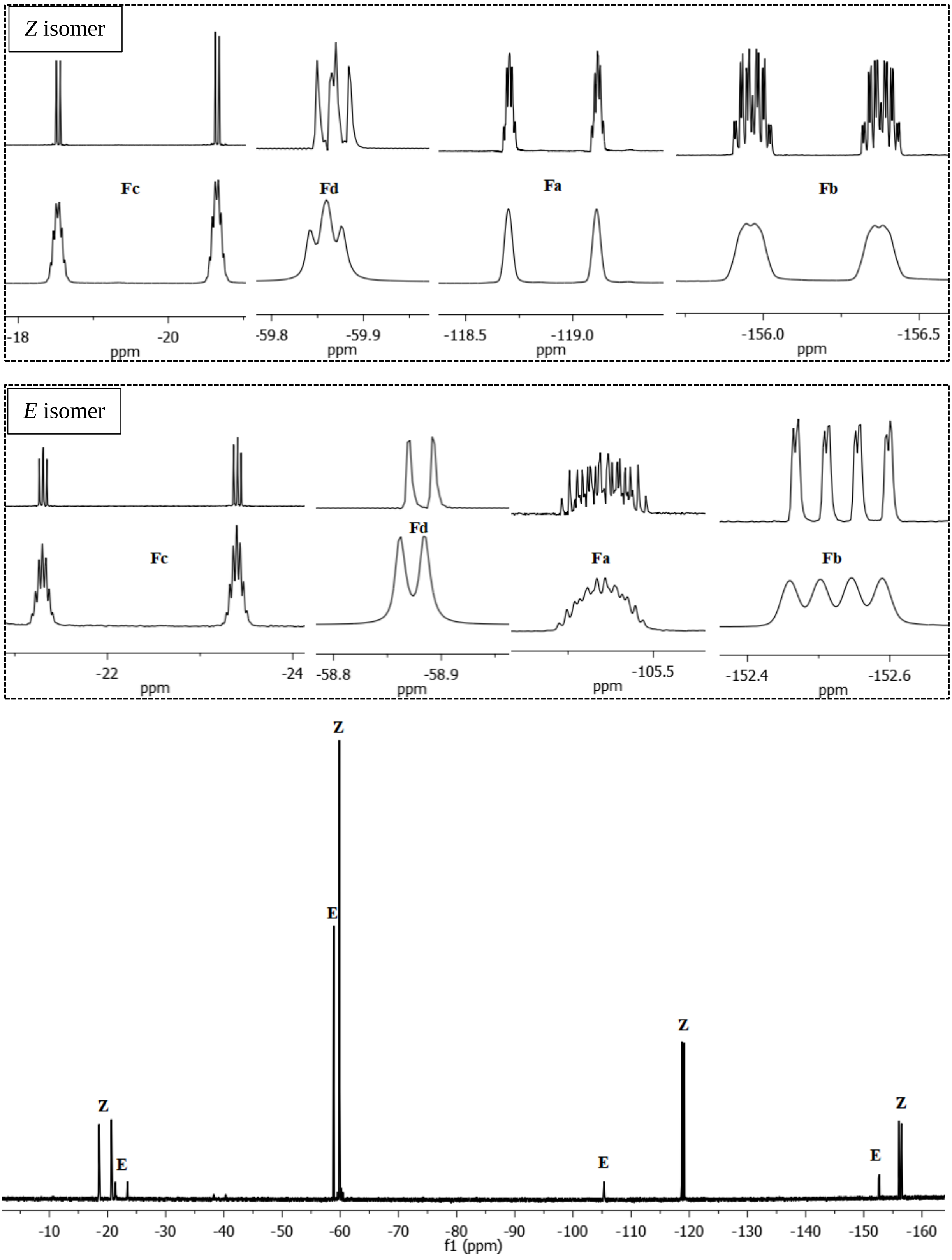

S18. ^{19}F NMR spectrum of the reaction of PEt_3 with excess of perfluoro (methyl vinyl ether) in C_6D_6 showing the formation of *Z/E*-(F_3CO)CF=CF($PFEt_3$). Zoomed in for each isomer with $^{19}F\{^1H\}$ NMR spectrum on the top.

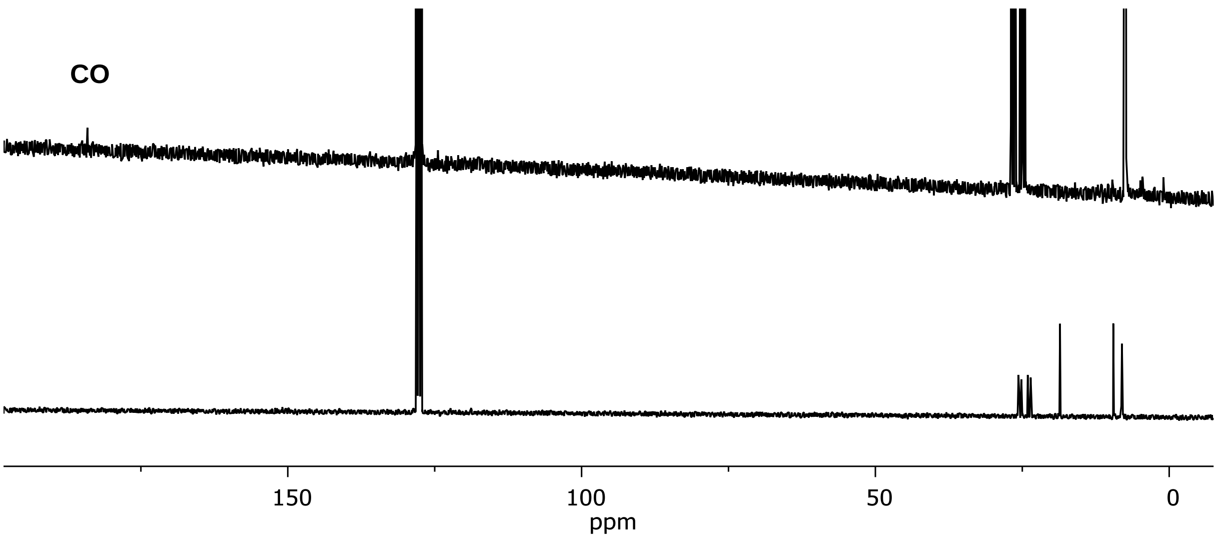

S19. $^{13}C\{^1H\}$ NMR spectrum of the reaction of PEt_3 with excess of perfluoro (methyl vinyl ether) in C_6D_6 showing the formation CO upon the decomposition of *Z/E*-$(F_3CO)CF{=}CF(PFEt_3)$.

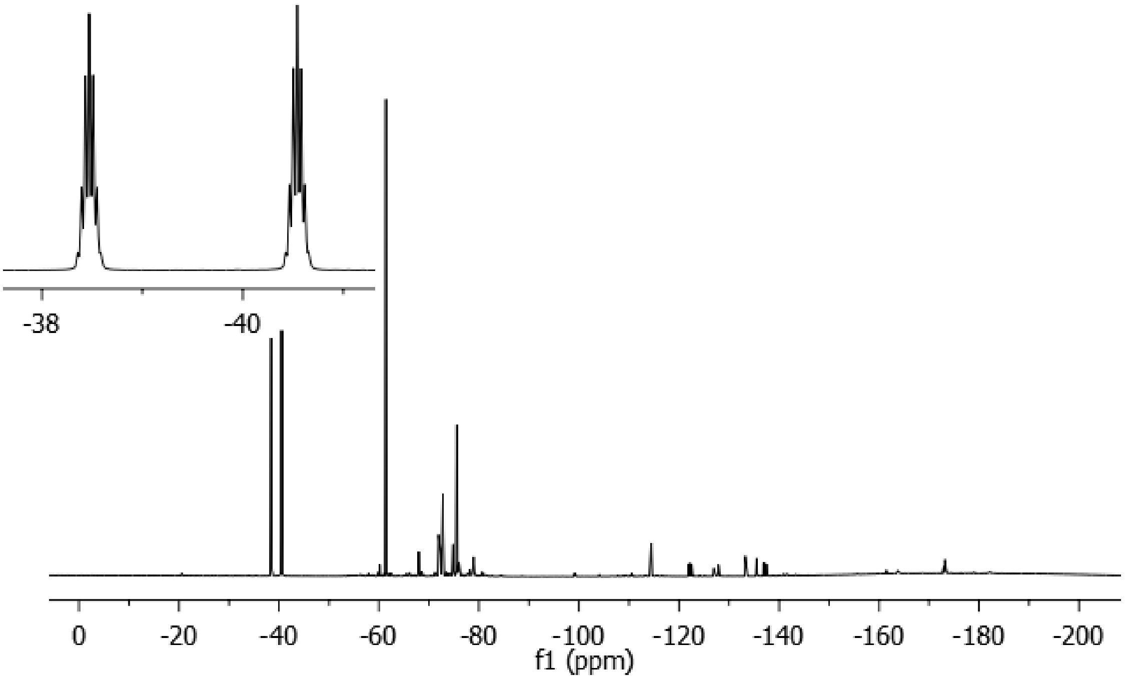

S20. ^{19}F NMR spectrum of the reaction of PEt_3 with excess of perfluoro (methyl vinyl ether) in C_6D_6 highlighting the formation Et_3PF_2.

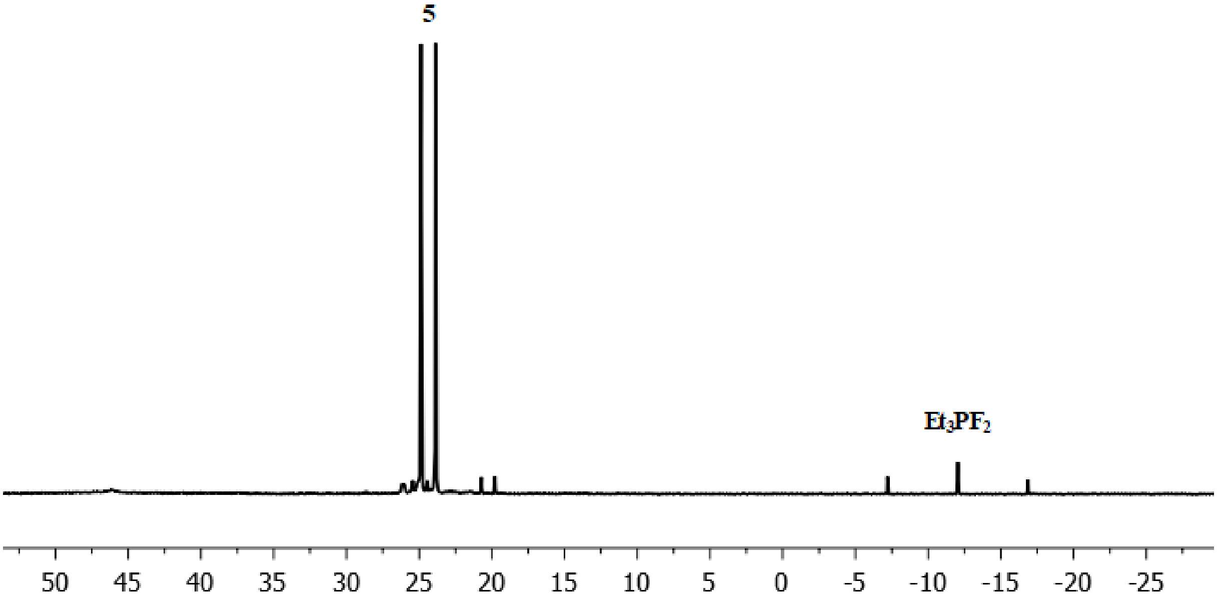

S21. $^{31}P\{^{1}H\}$ NMR spectrum of the reaction of complex **1** with excess of perfluoro (methyl vinyl ether) in C_6D_6 after the separation showing the formation of complex **5**.

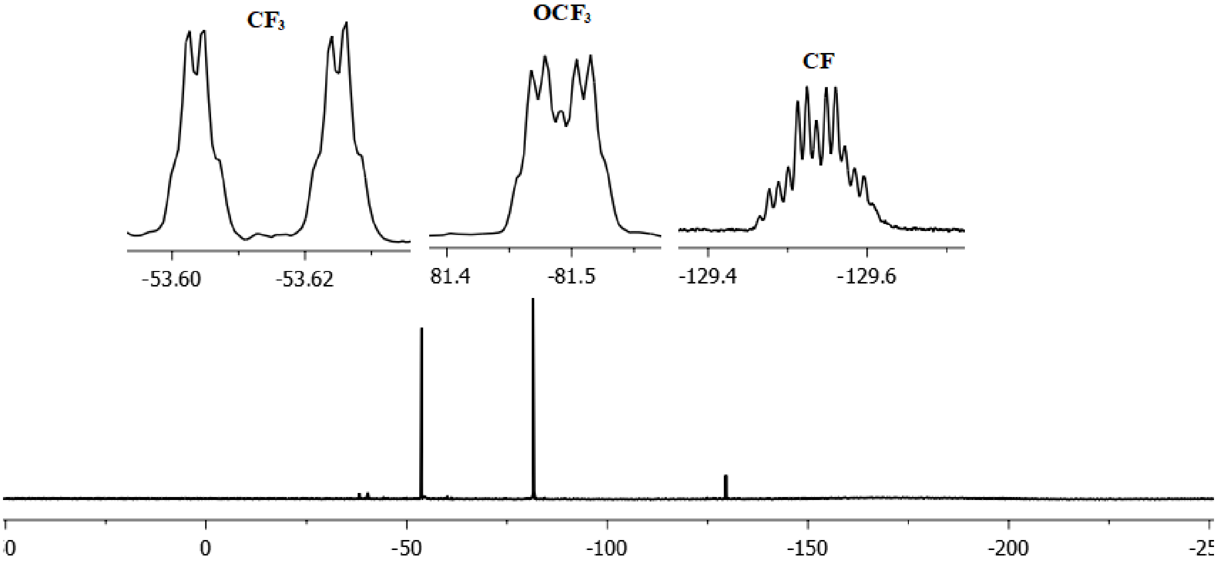

S22. ^{19}F NMR spectrum of the reaction of complex **1** with excess of perfluoro (methyl vinyl ether) in C_6D_6 after the separation showing the formation of complex **5**.

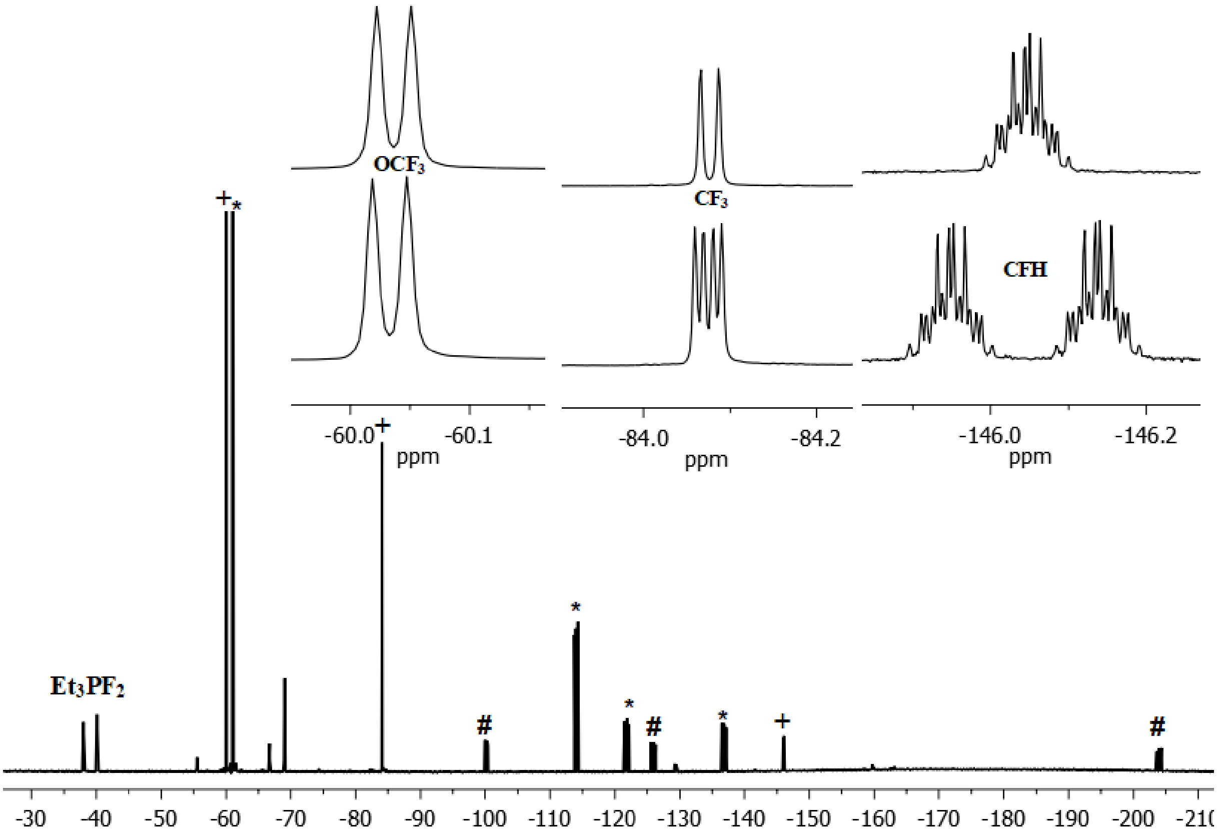

S23. ^{19}F NMR spectrum of the reaction of complex **1** with excess of perfluoro (methyl vinyl ether) in C_6D_6 after the separation highlighting the formation of OCF_3CF_3CFH (+) and $^{19}F\{^1H\}$ NMR spectrum (top) of it. * = *PMVE, # = trifluoroethylene.*

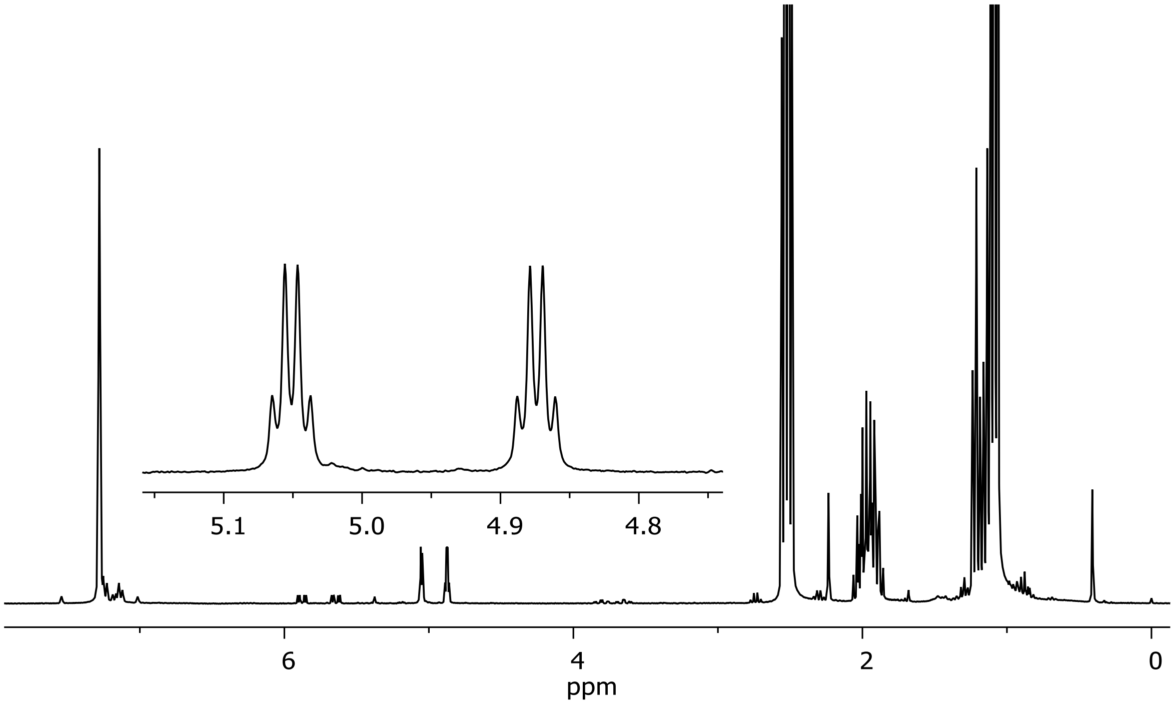

S24. ^1H NMR spectrum of the reaction of complex **1** with excess of perfluoro (methyl vinyl ether) in C_6D_6 after the separation highlighting the formation of OCF_3CF_3CFH.

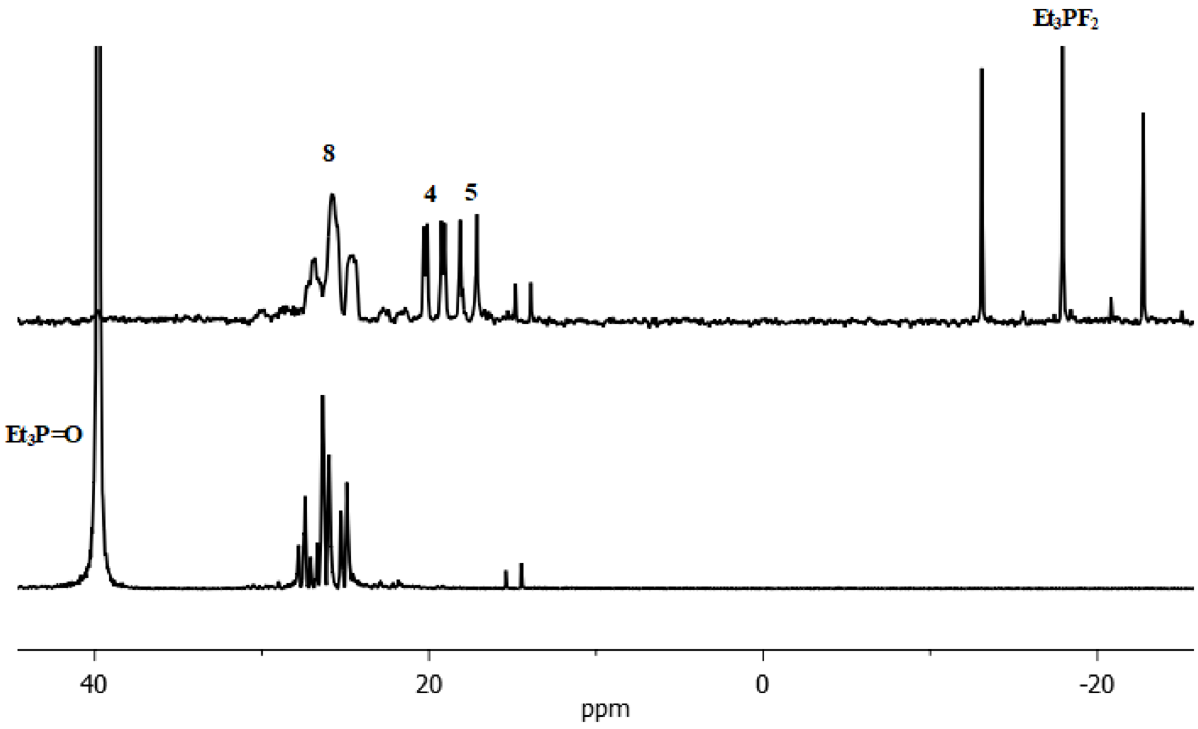

S25. $^{31}P\{^1H\}$ NMR spectrum of the reaction of complex **6** with excess of perfluoro (methyl vinyl ether) in C_6D_6 in presence of oxygen (bottom), and under inert condition (top) showing the formation of complex **8** and triethylphosphine oxide, and complexes **8**, **4**, **5** and difluorophosphorane, respectively.

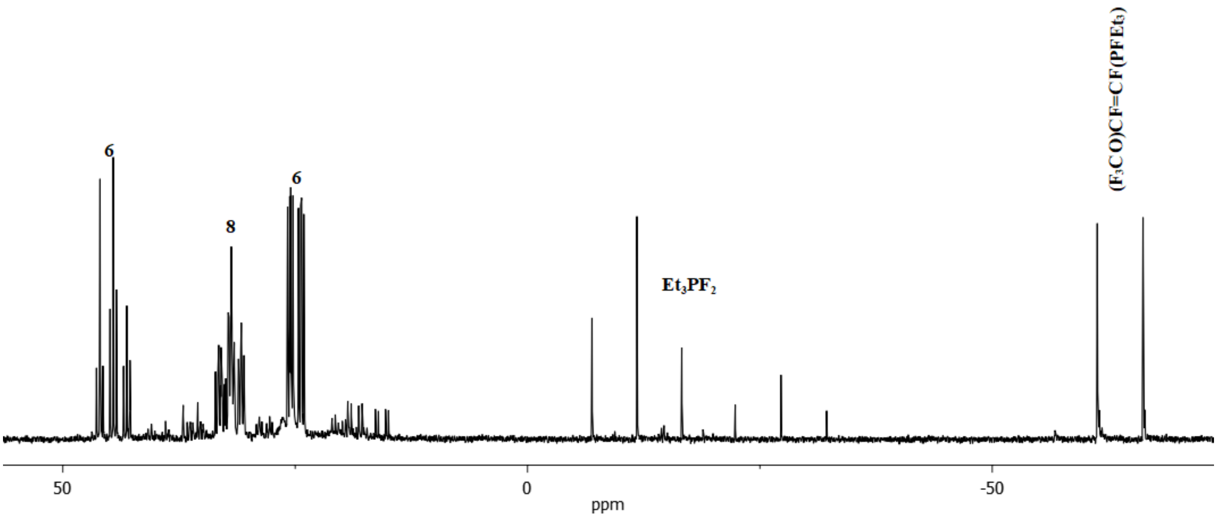

S26. $^{31}P\{^1H\}$ NMR spectrum of the reaction of complex **6** with excess of perfluoro (methyl vinyl ether) in C_6D_6 showing the formation of complex **8** and *Z*-(F_3CO)CF=CF($PFEt_3$) at the beginning of the reaction.

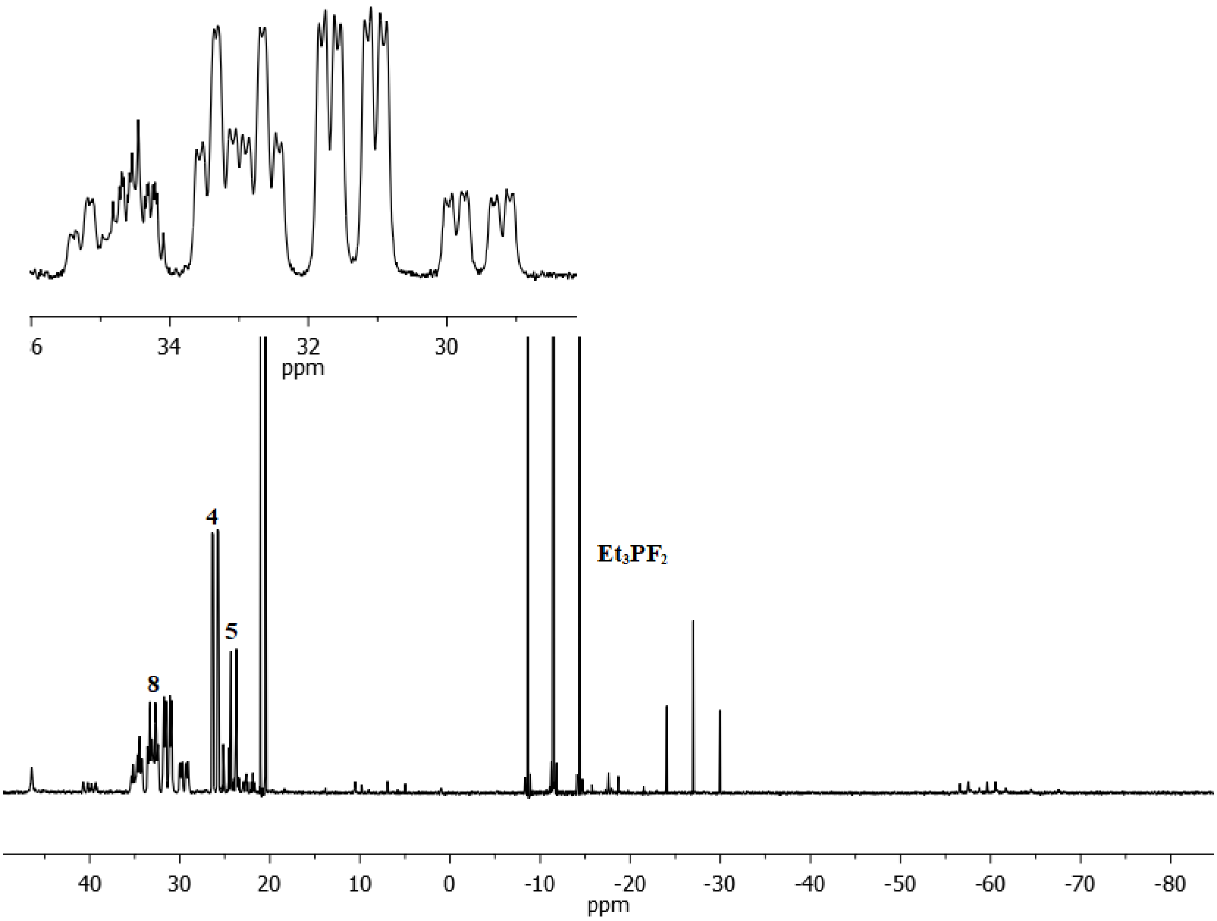

S27. $^{31}P\{^{1}H\}$ NMR spectrum (202.4 MHz) of the reaction of complex **6** with excess of perfluoro (methyl vinyl ether) in C_6D_6 showing the formation of complex **8,** as well as complexes **4** and **5** upon the decomposition of *Z*-(F_3CO)CF=CF($PFEt_3$).

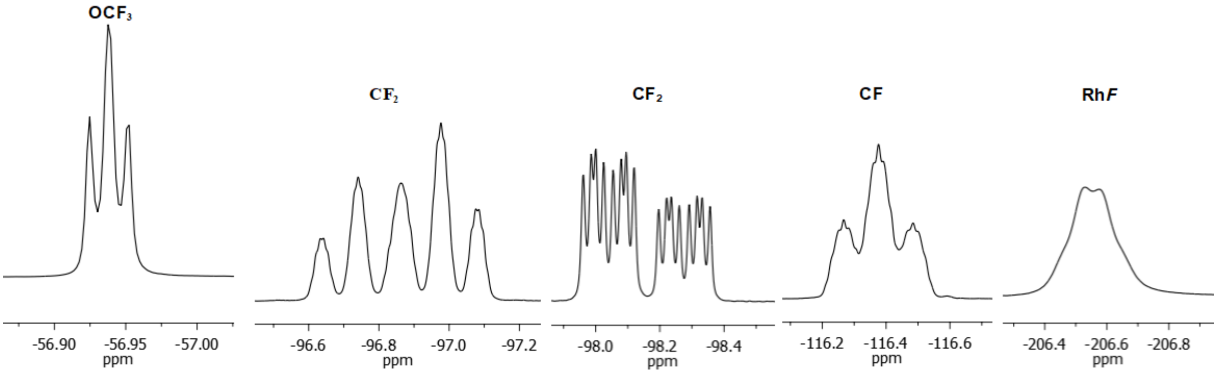

S28. Section of the ^{19}F NMR spectrum (470.6 MHz) of the reaction of complex **6** with excess of perfluoro (methyl vinyl ether) in C_6D_6 showing the formation of complex **8**.

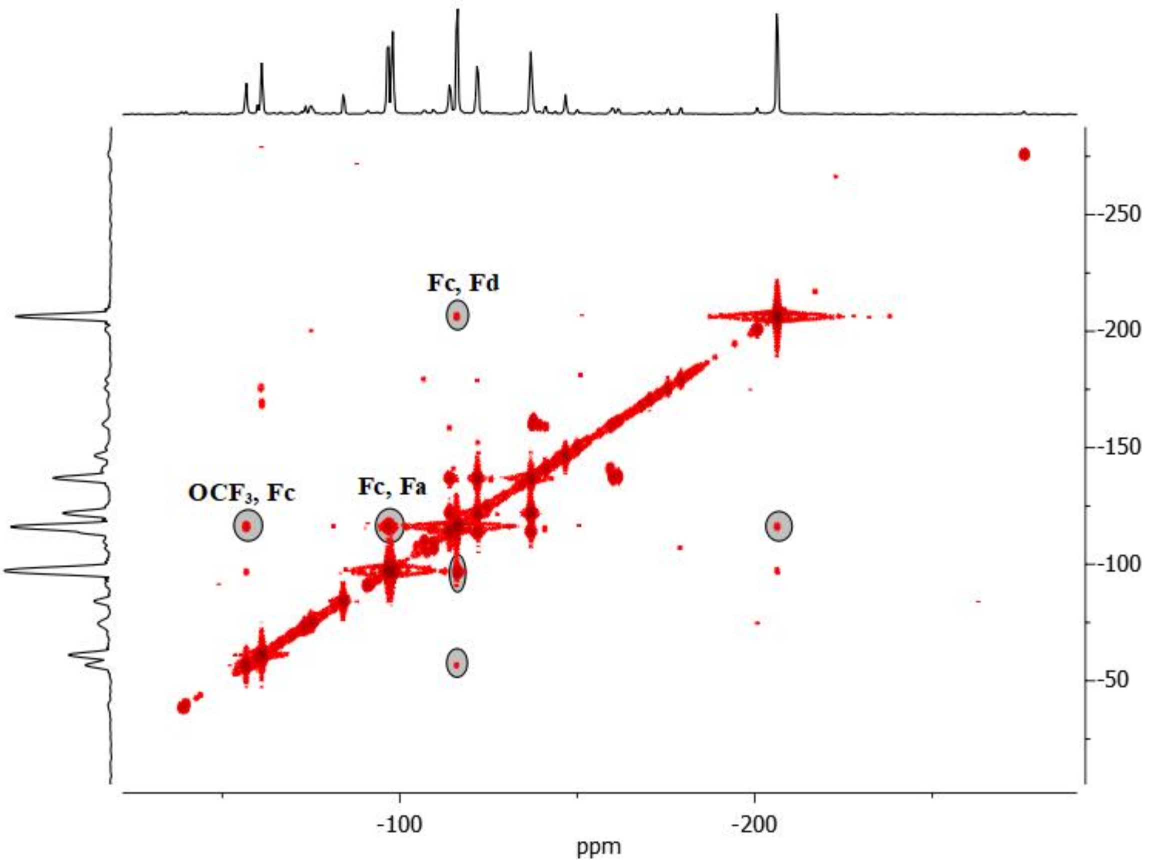

S29. ^{19}F-^{19}F COSY NMR spectrum of the reaction of complex **6** with excess of perfluoro (methyl vinyl ether) in C_6D_6 showing the formation of complex **8**.

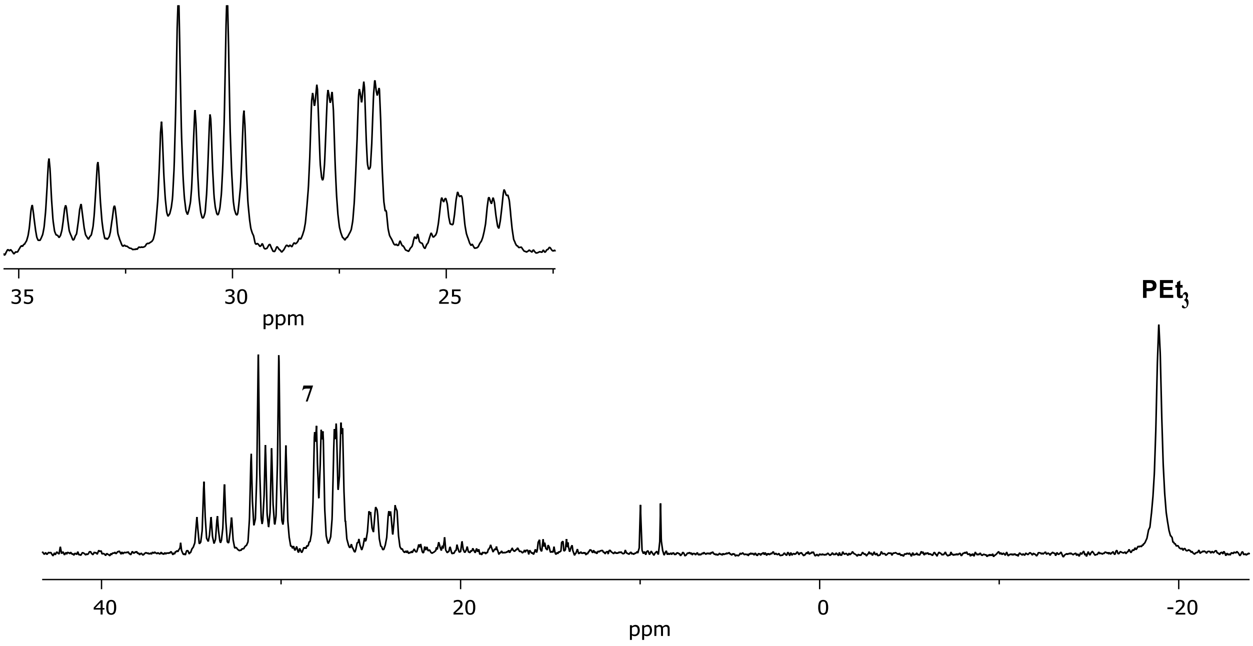

S30. ^{31}P{^{1}H} NMR spectrum of the reaction of complex **6** with excess of trifluoroethylene in C_6D_6 showing the formation of complex **7** and dissociation of the PEt_3.

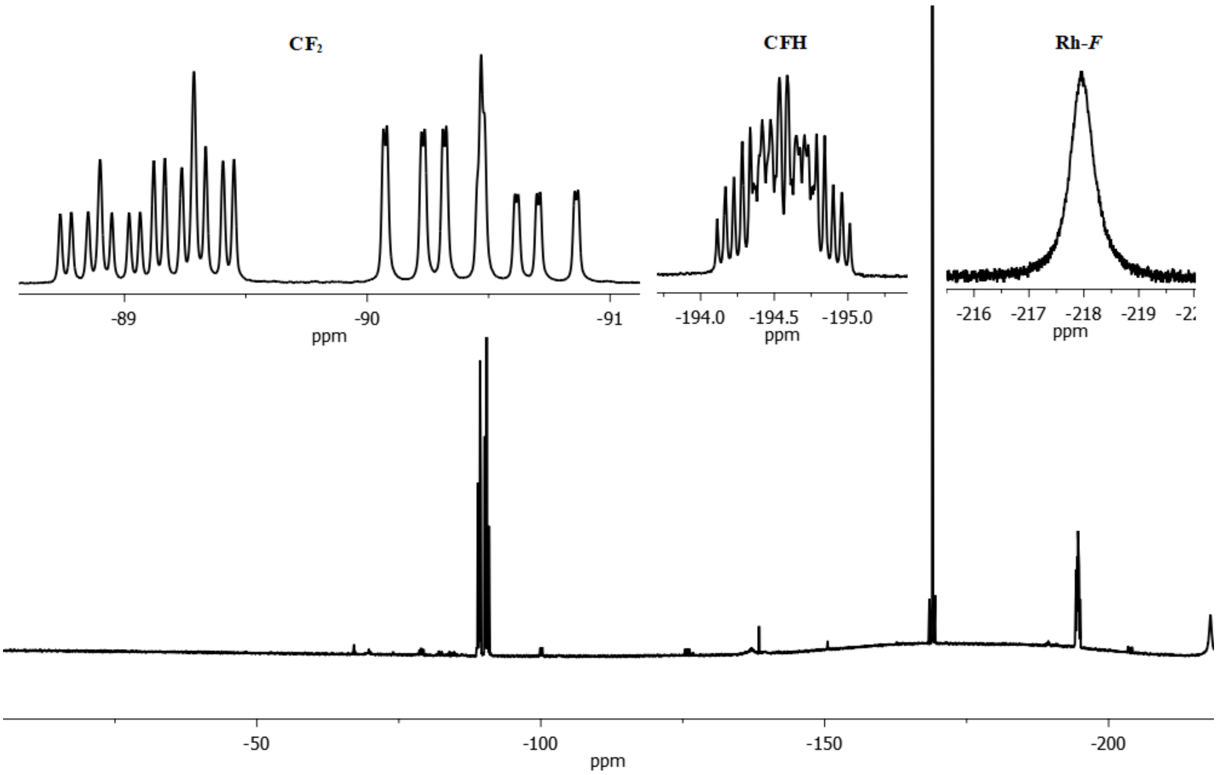

S31. ^{19}F NMR spectrum (470.6 MHz) of the reaction of complex **6** with excess of trifluoroethylene in C_6D_6 showing the formation of complex **7**.

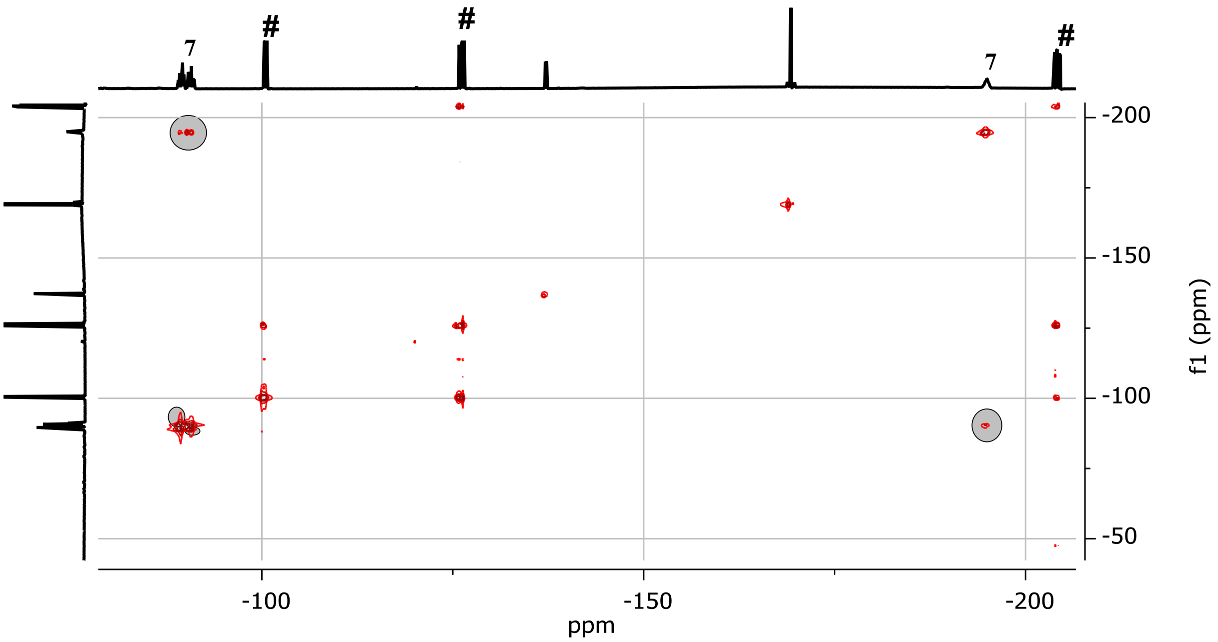

S32. Section of the ^{19}F-^{19}F COSY NMR spectrum of the reaction of complex **6** with excess of trifluoroethylene in C_6D_6 showing the formation of complex **7**. # = *trifluoroethylene.*

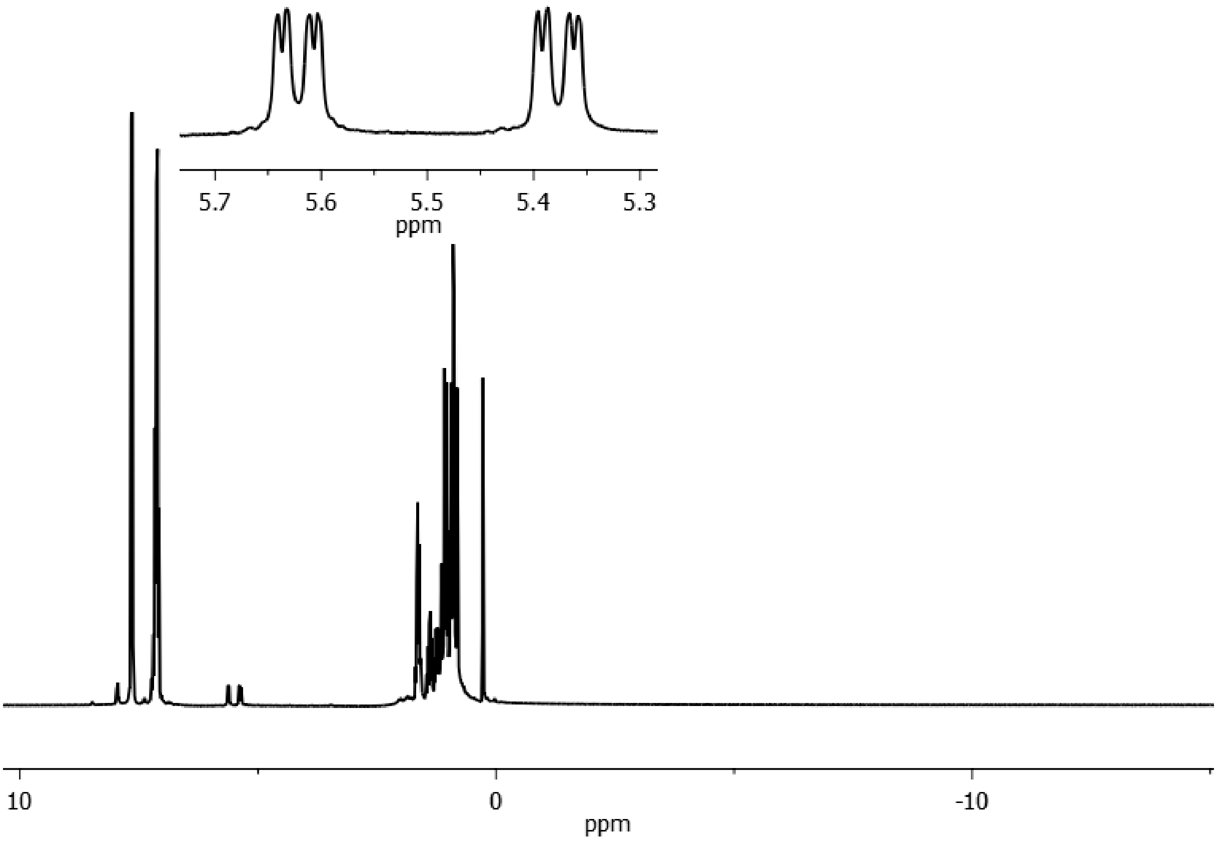

S33. ^{1}H NMR spectrum of the reaction of complex **1** with excess of trifluoroethylene in C_6D_6 showing the formation of complex **7**.

2. DFT Calculations

2.1 Computational details for geometry optimization of all the calculated complexes

DFT calculations for compounds **2***, **2'*** and **8** were performed using the Gaussian 16 (Revision A.03) program package[14] and the B3LYP functional, Rh was described on using a RECP with the associated cc-pvdz basis set.[15] For **8**, cc-pvtz basis sets were employed for all the other atoms. For **2*** and **2'***, cc-pvdz basis sets were employed for all C, P and O atoms, while cc-pvtz basis sets were employed for H and F atoms. For all calculations, dispersion effects were taken into account using the Grimme D3 dispersion correction with Becke-Johnson damping.[16] Frequency calculations were run for all stationary points to identify them as minima (no negative eigenvalues). Energies were corrected for zero-point energy.

2.2 Structure optimization of complexes 2*, 2'* and 8

Cartesian coordinates of all optimized structures:

Complex 2*: Energy: -2282.956007 [Hartree/Particle]

Rh	0.23755	-0.06301	-0.23642
P	-0.04946	2.26744	0.14086
C	1.26947	3.26699	0.99298
C	-1.54097	2.86360	1.06426
P	2.25096	-0.09172	-1.41316
C	3.23037	-1.63471	-1.11285
C	3.57119	1.21090	-1.29817
C	1.98269	-0.18037	-3.23880
C	-0.21329	3.20952	-1.44413
C	-0.38861	-1.94500	-0.63917
C	-1.44843	-1.23186	0.09227
O	-2.69024	-1.18702	-0.58627

F	-1.70187	-1.74112	1.37312
F	0.15281	-3.05926	-0.02765
F	-0.64675	-2.32407	-1.93428
C	-3.45750	-0.09401	-0.45231
F	-3.55153	0.35219	0.82228
F	-4.69317	-0.39938	-0.87673
F	-3.03307	0.96075	-1.19720
P	1.13054	-0.50868	1.95667
C	2.80361	0.18231	2.37372
C	1.34745	-2.25835	2.51415
C	0.09405	0.17806	3.32769
H	-0.41351	0.28210	-1.62921
H	1.29590	-1.00317	-3.43581
H	2.91924	-0.33168	-3.78008
H	1.50777	0.74289	-3.57082
H	2.56843	-2.48940	-1.24985
H	3.59249	-1.64636	-0.08492
H	4.08096	-1.70850	-1.79392
H	3.91601	1.29250	-0.26729
H	3.15422	2.17231	-1.60105
H	4.42015	0.97303	-1.94328
H	3.55937	-0.30845	1.76018
H	3.04316	0.01382	3.42575
H	2.83087	1.24989	2.16496

H	0.38023	-2.75481	2.49115
H	1.76286	-2.28516	3.52379
H	2.01110	-2.77836	1.82510
H	-0.92004	-0.19766	3.19875
H	0.07642	1.26587	3.26509
H	0.48026	-0.11793	4.30525
H	-2.43499	2.56751	0.52265
H	-1.51876	3.95035	1.17283
H	-1.57240	2.39846	2.04853
H	-1.06098	2.80405	-1.99484
H	0.68618	3.06043	-2.04217
H	-0.36006	4.27698	-1.26569
H	1.34709	2.96099	2.03649
H	1.03057	4.33225	0.95903
H	2.23114	3.09940	0.50930

Complex 2’*: Energy: -2282.951980 [Hartree/Particle]

Rh	-0.40366	-0.02814	-0.36724
P	-2.69901	-0.49135	-0.44245
C	-3.89340	-0.18913	0.95179
C	-3.02616	-2.26918	-0.83237
P	-0.46971	2.32877	-0.07042
C	0.83498	3.20421	0.91399
C	-1.99598	3.12289	0.63787
C	-0.32606	3.21660	-1.68671
C	-3.58745	0.35024	-1.83195
C	1.42853	-0.63475	-1.13875
C	0.43429	-1.71950	-1.10319
F	0.04769	-2.26177	-2.30348
F	0.73024	-2.79625	-0.27884
O	2.62296	-0.95442	-0.41910
F	1.79754	-0.20965	-2.40201
P	-0.11511	-0.67911	1.93050
C	-0.84358	0.44945	3.21138
C	1.57069	-0.96864	2.63226
C	-0.93207	-2.28428	2.36930
H	-0.66790	0.34390	-1.87752
C	3.48076	0.02641	-0.10386
F	4.44320	-0.50612	0.67151

F	2.91623	1.04513	0.60304
F	4.07904	0.59788	-1.16748
H	1.80160	3.03618	0.44546
H	0.87532	2.80511	1.92692
H	0.62805	4.27576	0.95620
H	-2.86486	2.82859	0.04921
H	-1.91608	4.21232	0.63277
H	-2.14080	2.78046	1.66289
H	-1.15928	2.92251	-2.32466
H	0.59621	2.89420	-2.16986
H	-0.32151	4.30102	-1.55603
H	-1.89446	0.62619	2.98528
H	-0.32054	1.40521	3.19184
H	-0.75701	0.01905	4.21125
H	2.08224	-1.70907	2.02001
H	1.49586	-1.32528	3.66150
H	2.14229	-0.04344	2.60535
H	-3.58834	-0.75014	1.83452
H	-4.90401	-0.49504	0.67187
H	-3.89739	0.87161	1.20246
H	-2.46687	-2.52525	-1.73160
H	-4.09119	-2.45893	-0.98293
H	-2.65362	-2.89033	-0.01886
H	-3.07355	0.10451	-2.76051

H	-3.54447	1.43064	-1.69368
H	-4.63131	0.03414	-1.88897
H	-0.53000	-3.05766	1.71746
H	-2.00672	-2.21378	2.20753
H	-0.74588	-2.54432	3.41328

Complex **8**

C	-0.02122	0.24713	1.81876
C	-0.14944	1.37847	0.93991
F	1.06475	0.16874	2.64492
F	-1.10309	-0.17473	2.54739
F	0.87134	2.31067	0.96098
O	-1.38122	2.05908	0.93551
C	-1.70588	2.76964	-0.17470
F	-2.96564	3.19458	-0.01524
F	-1.65576	2.04053	-1.31161
F	-0.91920	3.83972	-0.36259
Rh	0.15471	-0.33898	-0.06630
F	0.48180	-1.68605	-1.53254
P	-2.06718	-1.16765	-0.17121
C	-3.54765	-0.17142	0.33636
C	-2.19580	-2.71028	0.85310
P	2.48533	-0.07116	-0.32336
C	3.51137	1.09897	0.69188

C	2.75236	0.36399	-2.10422
C	5.00843	0.79624	0.79579
C	4.18190	0.39526	-2.64591
C	-0.94534	-3.58977	0.77540
C	-4.87803	-0.92556	0.38149
C	3.23838	-1.74629	-0.10978
C	3.07431	-2.28302	1.31442
C	-2.43990	-1.75746	-1.88440
C	-2.35653	-0.65063	-2.93624

3. References

1. D. D. Perrin and W. L. F. Armarego, *Purification of Laboratory Chemicals*, Butterworth/Heinemann, London/Oxford, 3rd edn., 1988.
2. T. Braun, D. Noveski, M. Ahijado and F. Wehmeier, *Dalton Transactions*, 2007, 3820-3825.
3. N. Pfister, T. Braun, P. Wittwer and M. Ahrens, *Z. Anorg. Allg. Chem.*, 2018, **644**, 1064-1070.
4. M. Talavera, C. N. von Hahmann, R. Müller, M. Ahrens, M. Kaupp and T. Braun, *Angew. Chem. Int. Ed.*, 2019, **58**, 10688-10692.
5. M. Talavera, C. N. von Hahmann, R. Muller, M. Ahrens, M. Kaupp and T. Braun, *Angew. Chem. Int. Ed. Engl.*, 2019, **58**, 10688-10692.
6. M. Talavera and T. Braun, *Chem. Eur. J.*, 2021, **27**, 11926-11934.
7. P. K. Sazonov, G. A. Artamkina, V. N. Khrustalev, M. Y. Antipin and I. P. Beletskaya, *J. Organomet. Chem.*, 2003, **681**, 59-69.
8. A. Foris, *Magn. Reson. Chem.*, 2004, **42**, 534-555.
9. U. Allwörden and G. V. Röschenthaler, *Chemiker-Zeitung*, 1988, **112**, 69-76.
10. A. L. Raza, M. F. Kuehnel, M. Talavera, M. Teltewskoi, M. Ahrens, P. Kläring, T. Braun and D. Lentz, *J. Fluorine Chem.*, 2018, **214**, 80-85.
11. D. Noveski, T. Braun, M. Schulte, B. Neumann and H.-G. Stammler, *Dalton Trans.*, 2003, DOI: 10.1039/b306635e, 4075-4083.
12. A. A. Facundo, A. Arévalo, G. Fundora-Galano, M. Flores-Álamo, E. Orgaz and J. J. García, *New J. Chem.*, 2019, **43**, 6897-6908.
13. J. Seravalli and S. W. Ragsdale, *Biochem.,* 2008, **47**, 6770-6781.
14. M. J. Frisch, G. W. Trucks, H. B. Schlegel, G. E. Scuseria, M. A. Robb, J. R. Cheeseman, G. Scalmani, V. Barone, G. A. Petersson, H. Nakatsuji, X. Li, M. Caricato, A. V. Marenich, J. Bloino, B. G. Janesko, R. Gomperts, B. Mennucci, H. P. Hratchian, J. V. Ortiz, A. F. Izmaylov, J. L. Sonnenberg, Williams, F. Ding, F. Lipparini, F. Egidi, J. Goings, B. Peng, A. Petrone, T. Henderson, D. Ranasinghe, V. G. Zakrzewski, J. Gao, N. Rega, G. Zheng, W. Liang, M. Hada, M. Ehara, K. Toyota, R. Fukuda, J. Hasegawa, M. Ishida, T. Nakajima, Y. Honda, O. Kitao, H. Nakai, T. Vreven, K. Throssell, J. A. Montgomery Jr., J. E. Peralta, F. Ogliaro, M. J. Bearpark, J. J. Heyd, E. N. Brothers, K. N. Kudin, V. N. Staroverov, T. A. Keith, R. Kobayashi, J. Normand, K. Raghavachari, A. P. Rendell, J. C. Burant, S. S. Iyengar, J. Tomasi, M. Cossi, J. M. Millam, M. Klene, C. Adamo, R. Cammi, J. W. Ochterski, R. L. Martin, K. Morokuma, O. Farkas, J. B. Foresman and D. J. Fox, *Journal*, 2016.
15. K. A. Peterson, D. Figgen, M. Dolg and H. Stoll, *J. Chem. Phys.*, 2007, **126**, 124101.
16. S. Grimme, S. Ehrlich and L. Goerigk, *J. Comput. Chem.*, 2011, **32**, 1456-1465.
17. K. A. Giffin, L. A. Pua, S. Piotrkowski, B. M. Gabidullin, I. Korobkov, R. P. Hughes, and R. T. Baker, *J. Am.Chem. Soc.*, 2017, **139**, 4075-4086.
